# An Unbiased Estimator of Gene Diversity with Improved Variance for Samples Containing Related and Inbred Individuals of any Ploidy

**DOI:** 10.1534/g3.116.037168

**Published:** 2016-12-30

**Authors:** Alexandre M. Harris, Michael DeGiorgio

**Affiliations:** * Department of Biology, Pennsylvania State University, University Park, Pennsylvania 16802; † Molecular, Cellular, and Integrative Biosciences at the Huck Institutes of the Life Sciences, Pennsylvania State University, University Park, Pennsylvania 16802; ‡ Institute for CyberScience, Pennsylvania State University, University Park, Pennsylvania 16802

**Keywords:** expected heterozygosity, identity state, inbreeding, locus-specific branch length, relatedness

## Abstract

Gene diversity, or expected heterozygosity (*H*), is a common statistic for assessing genetic variation within populations. Estimation of this statistic decreases in accuracy and precision when individuals are related or inbred, due to increased dependence among allele copies in the sample. The original unbiased estimator of expected heterozygosity underestimates true population diversity in samples containing relatives, as it only accounts for sample size. More recently, a general unbiased estimator of expected heterozygosity was developed that explicitly accounts for related and inbred individuals in samples. Though unbiased, this estimator’s variance is greater than that of the original estimator. To address this issue, we introduce a general unbiased estimator of gene diversity for samples containing related or inbred individuals, which employs the best linear unbiased estimator of allele frequencies, rather than the commonly used sample proportion. We examine the properties of this estimator, ${\tilde{H}}_{\text{BLUE}},$ relative to alternative estimators using simulations and theoretical predictions, and show that it predominantly has the smallest mean squared error relative to others. Further, we empirically assess the performance of ${\tilde{H}}_{\text{BLUE}}$ on a global human microsatellite dataset of 5795 individuals, from 267 populations, genotyped at 645 loci. Additionally, we show that the improved variance of ${\tilde{H}}_{\text{BLUE}}$ leads to improved estimates of the population differentiation statistic, $F_{\text{ST}},$ which employs measures of gene diversity within its calculation. Finally, we provide an R script, *BestHet*, to compute this estimator from genomic and pedigree data.

The gene diversity of a locus, also known as its expected heterozygosity (*H*), is a fundamental measure of genetic variation in a population, and describes the proportion of heterozygous genotypes expected under Hardy-Weinberg equilibrium (Nei 1973). Formally, gene diversity is the probability that a pair of randomly sampled allele copies from a population are different, and is computed as

$$H=1-\sum_{i=1}^{I}p_{i}^{2},$$ (1)

where *I* is the number of distinct alleles at a locus, and $p_{i}$ (i=1,2,…,I) is the frequency of allele *i* in the population.

For a sample without related or inbred individuals composed of *n* allele copies, an unbiased estimator of expected heterozygosity is (Nei and Roychoudhury 1974)

$$\hat{H}=\frac{n}{n-1}\left(1-\sum_{i=1}^{I}{\hat{p}}_{i}^{2}\right),$$ (2)

where ${\hat{p}}_{i}$ is the sample proportion of allele *i*. $\hat{H}$ is a biased estimator when inbred or related individuals are included in the sample (DeGiorgio and Rosenberg 2009). This result is based on the idea that, as the proportion of related individuals in the sample increases, the number of independent allele observations decreases.

When two alleles are drawn from a sample, one each from a pair of related individuals, there is a nonzero probability that they will be identical by descent (IBD), rather than just identical by state (Lange 2002). This IBD probability is known as the kinship coefficient, and is denoted by $\Phi_{jk}$ for a pair of individuals *j* and *k*. Thus, the observed diversity will be lower than the true value because a greater proportion of identical alleles are observed than for a sample in which there are no related individuals. DeGiorgio *et al.* (2010) developed an estimator of expected heterozygosity,

$$\tilde{H}=\frac{1}{1-{\bar{\Phi }}_{2}}\left(1-\sum_{i=1}^{I}{\hat{p}}_{i}^{2}\right),$$ (3)

which is unbiased for samples containing related and inbred individuals of any ploidy, and employs a weighted mean kinship coefficient ${\bar{\Phi }}_{2}$ as a bias correction factor. ${\bar{\Phi }}_{2}$ is the average of all kinship coefficients $\Phi_{jk}$ for every pair of individuals within the sample (see *Methods*). Further, DeGiorgio *et al.* (2010) derived the theoretical variance of $\tilde{H},$ as well as its approximate value for samples wherein individuals are related to no more than one other sampled individual.

As an alternative to the sample proportion (${\hat{p}}_{i}$), McPeek *et al.* (2004) introduced the best linear unbiased estimator (BLUE, denoted as ${\tilde{p}}_{i}$) of population allele frequency, which is an unbiased linear estimator with smaller variance than the unbiased linear estimator ${\hat{p}}_{i}.$ The BLUE incorporates the relatedness of individuals in the sample as a covariance matrix to define the weight of each observation. Simulations and analytical evaluation corroborating their result suggest that the mean squared error (MSE) of ${\tilde{p}}_{i}$ is always smaller than that of ${\hat{p}}_{i},$ and this difference is especially evident for samples with complex pedigrees.

Because ${\tilde{p}}_{i}$ has the smallest variance of any unbiased linear estimator of allele frequencies, we expect its low variance to translate to smaller variance of gene diversity statistics that use ${\tilde{p}}_{i}.$ We developed such a statistic, termed ${\tilde{H}}_{\text{BLUE}},$ that is an unbiased estimator of expected heterozygosity in samples containing related and inbred individuals of arbitrary ploidy. Through simulations, analytical predictions, and empirical assessments, we compare the performance of ${\tilde{H}}_{\text{BLUE}}$ to that of $\tilde{H}$ and $\hat{H}$ for samples containing related individuals of various types across different ploidy and inbreeding status. Additionally, we derive the variance of any measure of expected heterozygosity that uses unbiased linear estimators of allele frequencies. We find that the increased precision of allele frequency estimates transfers to our unbiased estimator, yielding values for MSE invariably equal to or smaller than those of $\tilde{H},$ while occasionally exceeding the precision of $\hat{H}.$ The improved properties of ${\tilde{H}}_{\text{BLUE}}$ translate to its applications as well, which we demonstrate in the calculation of the population differentiation statistic, $F_{\text{ST}}$ (Wright 1951). $F_{\text{ST}}$ can be written in terms of intrapopulation and interpopulation gene diversity as (Hudson *et al.* 1992)

$$F_{\text{ST}}=\frac{H_{12}-\frac{1}{2}\left(H_{1}+H_{2}\right)}{H_{12}},$$ (4)

where $H_{1}$ and $H_{2}$ are the values of expected heterozygosity within each of two compared populations, and $H_{12}$ is the expected heterozygosity between them.

## Methods

Consider a locus with *I* distinct alleles in a sample of *n* individuals. Let $X_{k}^{\left(i\right)}$ denote the fraction of alleles at the locus in individual *k* that are of type *i*, i=1,2,…,I. An unbiased linear estimator of population allele frequencies $p_{i},$ denoted by ${\overset{˘}{p}}_{i},$ is defined as

$${\overset{˘}{p}}_{i}=\sum_{k=1}^{n}w_{k}X_{k}^{\left(i\right)},$$ (5)

where $w_{k},$ $0\leq w_{k}\leq 1,$ is the weight of individual *k*, k=1,2,…,n, and $\sum_{k=1}^{n}w_{k}=1.$ Formally, we have that

$$X_{k}^{\left(i\right)}=\frac{1}{m_{k}}\sum_{t=1}^{m_{k}}A_{kt}^{\left(i\right)},$$

where $A_{kt}^{\left(i\right)}$ is an indicator random variable whose value is 1 if allele *t* of individual *k* is of type *i*, and zero otherwise, and where $m_{k}$ is the ploidy of individual *k*. As an example, if individual *k* were diploid at the locus, then $m_{k}=2.$ Taking the expectation of ${\overset{˘}{p}}_{i},$

$$\begin{array}{c} E\left[{\overset{˘}{p}}_{i}\right]=\sum_{k=1}^{n}\frac{w_{k}}{m_{k}}\sum_{t=1}^{m_{k}}E\left[A_{kt}^{\left(i\right)}\right]\\ =\sum_{k=1}^{n}\frac{w_{k}}{m_{k}}\sum_{t=1}^{m_{k}}p_{i}\\ =p_{i}, \end{array}$$

shows that it is an unbiased estimator of $p_{i}.$

### Unbiased estimation of gene diversity using unbiased linear estimators of allele frequencies

In this section, we construct an unbiased estimator, $\overset{˘}{H},$ of expected heterozygosity that uses a general unbiased linear estimator, ${\overset{˘}{p}}_{i},$ of allele frequency $p_{i}$ (Proposition 1). We then show that the unbiased estimator, $\tilde{H},$ of DeGiorgio *et al.* (2010) follows as a corollary, assuming that ${\overset{˘}{p}}_{i}={\hat{p}}_{i},$ the sample proportion allele frequency estimator (Corollary 2). We then derive a new estimator, ${\tilde{H}}_{\text{BLUE}},$ also as a corollary, assuming that ${\overset{˘}{p}}_{i}={\tilde{p}}_{i},$ the BLUE of allele frequency (Corollary 3).

#### Proposition 1:

Consider a locus with *I* distinct alleles and parametric allele frequencies $p_{i}\in \left[0,1\right],$ i=1,2,…,I, and $\sum_{i=1}^{I}p_{i}=1.$ For a sample of size *n* individuals of any ploidy, inbreeding status, and relatedness,

$$\overset{˘}{H}=\frac{1}{1-\rho_{2}}\left(1-\sum_{i=1}^{I}{\overset{˘}{p}}_{i}^{2}\right)$$ (6)

is an unbiased estimator of expected heterozygosity, where

$$\rho_{2}=\sum_{j=1}^{n}\sum_{k=1}^{n}w_{j}w_{k}\Phi_{jk}$$

is a weighted mean kinship coefficient of the sample for all pairs of individuals in the sample, and where $w_{k},$ k=1,2,…,n, is the weight for individual *k*. The proof of Proposition 1 is found in the Appendix.

From ${\overset{˘}{p}}_{i},$ the sample proportion estimator ${\hat{p}}_{i}$ of allele frequency *i*, i=1,2,…,I, is recovered when $w_{k}=m_{k}/\sum_{j=1}^{n}m_{j}$ for individual *k*, k=1,2,…,n, leading to

$${\hat{p}}_{i}=\sum_{k=1}^{n}\frac{m_{k}}{\sum_{j=1}^{n}m_{j}}X_{k}^{\left(i\right)}.$$

Here, each individual is weighted by its contribution to the number of allele copies in the sample.

#### Corollary 2:

Consider a locus with *I* distinct alleles and parametric allele frequencies $p_{i}\in \left[0,1\right],$ i=1,2,…,I, and $\sum_{i=1}^{I}p_{i}=1.$ For a sample of size *n* individuals of any ploidy, inbreeding status, and relatedness,

$$\tilde{H}=\frac{1}{1-{\bar{\Phi }}_{2}}\left(1-\sum_{i=1}^{I}{\hat{p}}_{i}^{2}\right)$$ (7)

is an unbiased estimator of expected heterozygosity, where

$${\hat{p}}_{i}=\sum_{k=1}^{n}\frac{m_{k}}{\sum_{j=1}^{n}m_{j}}X_{k}^{\left(i\right)}$$

is the sample proportion estimator of allele frequency *i*, where

$${\bar{\Phi }}_{2}=\sum_{j=1}^{n}\sum_{k=1}^{n}\frac{m_{j}}{\sum_{x=1}^{n}m_{x}}\frac{m_{k}}{\sum_{y=1}^{n}m_{y}}\Phi_{jk}$$

is a weighted mean kinship coefficient of the sample for all pairs of individuals, and where $m_{k},$ k=1,2,…,n, is the ploidy for individual *k*. The proof of Corollary 2 is found in the Appendix.

It may be beneficial to apply an unbiased linear estimator of allele frequencies that has minimum variance. McPeek *et al.* (2004) introduced the BLUE of allele frequencies, which we formally define here. We will use the BLUE of allele frequencies to construct a new unbiased estimator of gene diversity that would ideally have improved variance over other estimators. Let K be an n×n symmetric matrix of kinship coefficients, with $K_{jk}=\Phi_{jk}.$ The BLUE (${\tilde{p}}_{i}$) of allele frequency is obtained when $w_{k}=\frac{\sum_{j=1}^{n}{\left(K^{-1}\right)}_{jk}}{1^{T}K^{-1}1},$ yielding

$${\tilde{p}}_{i}=\sum_{k=1}^{n}\frac{\sum_{j=1}^{n}{\left(K^{-1}\right)}_{jk}}{1^{T}K^{-1}1}X_{k}^{\left(i\right)},$$

where $K^{-1}$ denotes the inverse matrix of K, **1** is a column vector of *n* elements with all entries equal to 1, and $1^{T}$ is the transpose of **1**.

#### Corollary 3:

Consider a locus with *I* distinct alleles, and parametric allele frequencies $p_{i}\in \left[0,1\right],$ i=1,2,…,I, and $\sum_{i=1}^{I}p_{i}=1.$ For a sample of size *n* individuals of any ploidy, inbreeding status, and relatedness,

$${\tilde{H}}_{\text{BLUE}}=\frac{1}{1-\kappa_{2}}\left(1-\sum_{i=1}^{I}{\tilde{p}}_{i}^{2}\right)$$ (8)

is an unbiased estimator of expected heterozygosity, where

$${\tilde{p}}_{i}=\sum_{k=1}^{n}\frac{\sum_{j=1}^{n}{\left(K^{-1}\right)}_{jk}}{1^{T}K^{-1}1}X_{k}^{\left(i\right)}$$

is the BLUE of allele frequencies, and where

$$\kappa_{2}=\sum_{j=1}^{n}\sum_{k=1}^{n}\frac{\sum_{x=1}^{n}{\left(K^{-1}\right)}_{xj}}{1^{T}K^{-1}1}\frac{\sum_{y=1}^{n}{\left(K^{-1}\right)}_{yk}}{1^{T}K^{-1}1}\Phi_{jk}$$

is a weighted mean kinship coefficient of the sample for all pairs of individuals. The proof of Corollary 3 is found in the Appendix.

### Variance of H estimators using unbiased linear estimators of allele frequencies

We now derive the equation (Proposition 4) describing the variance of the unbiased estimator $\overset{˘}{H},$ which takes ${\overset{˘}{p}}_{i}$ as the unbiased linear estimate of population allele frequency $p_{i}.$ This value depends on the weighted mean kinship coefficients of the sample for all pairs, trios, quartets, and pairs of pairs of individuals in the sample, defined as

$$\begin{array}{l} \rho_{2}=\sum_{j=1}^{n}\sum_{k=1}^{n}w_{j}w_{k}\Phi_{jk}\\ \rho_{3}=\sum_{j=1}^{n}\sum_{k=1}^{n}\sum_{j^{\prime }=1}^{n}w_{j}w_{k}w_{j^{\prime }}\Phi_{jkj^{\prime }}\\ \rho_{4}=\sum_{j=1}^{n}\sum_{k=1}^{n}\sum_{j^{\prime }=1}^{n}\sum_{k^{\prime }=1}^{n}w_{j}w_{k}w_{j^{\prime }}w_{k^{\prime }}\Phi_{jkj^{\prime }k^{\prime }}\\ \rho_{2,2}=\sum_{j=1}^{n}\sum_{k=1}^{n}\sum_{j^{\prime }=1}^{n}\sum_{k^{\prime }=1}^{n}w_{j}w_{k}w_{j^{\prime }}w_{k^{\prime }}\Phi_{jk,j^{\prime }k^{\prime }}. \end{array}$$

Here, $\Phi_{jkj^{\prime }}$ is the probability that three randomly sampled alleles, one each from individuals *j*, *k*, and $j^{\prime },$ are IBD. $\Phi_{jkj^{\prime }k^{\prime }}$ is the probability that four randomly sampled alleles, one each from individuals *j*, *k*, $j^{\prime },$ and $k^{\prime },$ are IBD. Finally, $\Phi_{jk,j^{\prime }k^{\prime }}$ is the joint probability that two randomly sampled alleles, one each from individuals *j* and *k* are IBD, and two randomly sampled alleles, one each from individuals $j^{\prime }$ and $k^{\prime },$ are IBD. Note that individuals *j*, *k*, $j^{\prime },$ and $k^{\prime }$ are not necessarily distinct. The variances of $\tilde{H}$ and of ${\tilde{H}}_{\text{BLUE}}$ follow as Corollaries 7 and 8, once again differing only in the weight of a sampled individual in the mean kinship coefficient calculation.

#### Proposition 4:

Consider a locus with *I* distinct alleles and parametric allele frequencies $p_{i}\in \left[0,1\right],$ i=1,2,…,I, and $\sum_{i=1}^{I}p_{i}=1.$ For a sample of size *n* individuals of any ploidy, inbreeding status, and relatedness,

$$\text{Var}\left[\overset{˘}{H}\right]=\frac{1}{{\left(1-\rho_{2}\right)}^{2}}\text{Var}\left[1-\sum_{i=1}^{I}{\overset{˘}{p}}_{i}^{2}\right]$$ (9)

is the variance of the unbiased estimator of expected heterozygosity $\overset{˘}{H},$ where $\rho_{2}=\sum_{j=1}^{n}\sum_{k=1}^{n}w_{j}w_{k}\Phi_{jk}$ is a weighted mean kinship coefficient of the sample, and where $w_{k}$ for k=1,2,…,n is the weight of individual *k*. Further, we have

$$\begin{array}{c} \text{Var}\left[1-\sum_{i=1}^{I}{\overset{˘}{p}}_{i}^{2}\right]=\rho_{2,2}-\rho_{2}^{2}+2\left(\rho_{2}^{2}-\rho_{4}\right)\sum_{i=1}^{I}p_{i}^{2}+4\left(2\rho_{4}+\rho_{2}-2\rho_{3}-\rho_{2,2}\right)\sum_{i=1}^{I}p_{i}^{3}\\ +\left(3\rho_{2,2}+8\rho_{3}-6\rho_{4}-4\rho_{2}-\rho_{2}^{2}\right){\left(\sum_{i=1}^{I}p_{i}^{2}\right)}^{2}. \end{array}$$ (10)

The proof of Equation 10 is presented for the specific case of $\text{Var}\left[1-\sum_{i=1}^{I}{\hat{p}}_{i}^{2}\right]$ in Appendix B of DeGiorgio *et al.* (2010), where ${\hat{p}}_{i}$ is substituted for ${\overset{˘}{p}}_{i},$ and ${\bar{\Phi }}_{2},$ ${\bar{\Phi }}_{3},$ and ${\bar{\Phi }}_{4},$ and ${\bar{\Phi }}_{2,2}$ coefficients are substituted for $\rho_{2},$ $\rho_{3},$ $\rho_{4},$ and $\rho_{2,2}$ coefficients, respectively. We provide an abbreviated version of this proof for the general case in the Appendix. Further, the approximate value of Equation 10 for samples wherein no individual is related to more than one other is

$$\text{Var}\left[1-\sum_{i=1}^{I}{\overset{˘}{p}}_{i}^{2}\right]\approx 4\rho_{2}\left[\sum_{i=1}^{I}p_{i}^{3}-{\left(\sum_{i=1}^{I}p_{i}^{2}\right)}^{2}\right].$$ (11)

For this simplifying case, the terms $\rho_{3},$ $\rho_{4},$ $\rho_{2,2},$ and $\rho_{2}^{2}$ are negligible compared to $\rho_{2}.$

In the Appendix, we reintroduce the definition of $\text{Var}\left[\tilde{H}\right]$ from DeGiorgio *et al.* (2010) (Corollary 7), and then define $\text{Var}\left[{\tilde{H}}_{\text{BLUE}}\right]$ (Corollary 8), both of which take the form illustrated in Proposition 4. As demonstrated by DeGiorgio *et al.* (2010), the mean kinship coefficients composing Equation 10 derive from the relationship between the 15 identity states available to four alleles (Gillois 1965; Cockerham 1971), and the coefficients of kinship between pairs, trios, quartets, and pairs of pairs of alleles within those four.

### Bias of $\hat{\text{H}}$ for samples containing related or inbred individuals

Here, we briefly derive an equation (Equation 12) within Proposition 5 that describes the bias of $\hat{H},$ which we display in the left panels of Supplemental Material, Figure S1A and Figure S2A. We include Corollaries 9 and 10 to Proposition 5 within the Appendix for specific cases of bias derived from ${\hat{p}}_{i}$-based and ${\tilde{p}}_{i}$-based estimations, respectively. We also note that Equation A10 of Corollary 9 represents the form of the bias typically encountered in applications of $\hat{H},$ as well as in all of our experimental scenarios.

#### Proposition 5:

Consider a locus with *I* distinct alleles and parametric allele frequencies $p_{i}\in \left[0,1\right],$ i=1,2,…,I, and $\sum_{i=1}^{I}p_{i}=1.$ For a sample of *n* possibly related or inbred individuals, the bias of the estimator of expected heterozygosity $\hat{H}$ changes with the true locus expected heterozygosity such that

$$\text{Bias}\left[\hat{H}\left({\overset{˘}{p}}_{i}\right)\right]=\frac{1-n\rho_{2}}{n-1}H,$$ (12)

where

$$\hat{H}\left({\overset{˘}{p}}_{i}\right)=\frac{n}{n-1}\left(1-\sum_{i=1}^{I}{\overset{˘}{p}}_{i}^{2}\right).$$ (13)

#### Proof:

We begin by substituting Equation 6 into Equation 13 such that

$$\hat{H}\left({\overset{˘}{p}}_{i}\right)=\frac{n\left(1-\rho_{2}\right)}{n-1}\overset{˘}{H},$$

and

$$E\left[\hat{H}\left({\overset{˘}{p}}_{i}\right)\right]=\frac{n\left(1-\rho_{2}\right)}{n-1}H.$$

From the definition of bias,

$$\begin{array}{c} \text{Bias}\left[\hat{H}\left({\overset{˘}{p}}_{i}\right)\right]=E\left[\hat{H}\left({\overset{˘}{p}}_{i}\right)\right]-H\\ =\frac{1-n\rho_{2}}{n-1}H. \end{array}$$ □

### Variance of ${\text{F}}_{\text{ST}}$ estimators using unbiased linear estimators of allele frequencies

Because the population differentiation statistic $F_{\text{ST}}$ (Wright 1951) can be defined in terms of expected heterozygosities, it is possible to theoretically evaluate its approximate variance. A general estimator of $F_{\text{ST}}$ can be written as

$${\overset{˘}{F}}_{\text{ST}}=\frac{{\overset{˘}{H}}_{12}-\frac{1}{2}\left({\overset{˘}{H}}_{1}+{\overset{˘}{H}}_{2}\right)}{{\overset{˘}{H}}_{12}},$$ (14)

where ${\overset{˘}{H}}_{12}$ is an unbiased estimator for the expected heterozygosity between a pair of sampled populations, numbered 1 and 2, defined as ${\overset{˘}{H}}_{12}=1-\sum_{i=1}^{I}{\overset{˘}{p}}_{i}{\overset{˘}{q}}_{i}$ (where ${\overset{˘}{q}}_{i}$ is a linear unbiased estimator of the frequency of allele *i* in population 2, analogous to ${\overset{˘}{p}}_{i}$ in population 1), while ${\overset{˘}{H}}_{1}$ and ${\overset{˘}{H}}_{2}$ are the within-population expected heterozygosities for populations 1 and 2, respectively. Referring to the numerator as *x*, and the denominator as *y*, we can write the expression for an approximation of the variance of a ratio as

$$\text{Var}\left[\frac{x}{y}\right]\approx \frac{{\left(E\left[x\right]\right)}^{2}}{{\left(E\left[y\right]\right)}^{2}}\left[\frac{\text{Var}\left[x\right]}{{\left(E\left[x\right]\right)}^{2}}+\frac{\text{Var}\left[y\right]}{{\left(E\left[y\right]\right)}^{2}}-2\frac{\text{Cov}\left[x,y\right]}{E\left[x\right]E\left[y\right]}\right],$$ (15)

following the definition for the approximate variance of a ratio (Wolter 2007).

#### Proposition 6:

Consider a locus with *I* distinct alleles across two populations and parametric allele frequencies $p_{i}\in \left[0,1\right],$ i=1,2,…,I, and $\sum_{i=1}^{I}p_{i}=1$ for population 1, and $q_{i}\in \left[0,1\right],$ i=1,2,…,I, and $\sum_{i=1}^{I}q_{i}=1$ for population 2. For samples of size $n_{1}$ and $n_{2}$ individuals from populations 1 and 2, respectively, each with individuals of any ploidy, inbreeding status, and relatedness, the variance of the population differentiation statistic calculated from their respective expected heterozygosities is approximated as

$$\text{Var}\left[{\overset{˘}{F}}_{\text{ST}}\right]\approx \frac{{\left[H_{12}-\frac{1}{2}\left(H_{1}+H_{2}\right)\right]}^{2}}{H_{12}^{2}}\times \left[\frac{\text{Var}\left[{\overset{˘}{H}}_{12}-\frac{1}{2}\left({\overset{˘}{H}}_{1}+{\overset{˘}{H}}_{2}\right)\right]}{{\left[H_{12}-\frac{1}{2}\left(H_{1}+H_{2}\right)\right]}^{2}}+\frac{\text{Var}\left[{\overset{˘}{H}}_{12}\right]}{H_{12}^{2}}-2\frac{\text{Cov}\left[{\overset{˘}{H}}_{12}-\frac{1}{2}\left({\overset{˘}{H}}_{1}+{\overset{˘}{H}}_{2}\right),{\overset{˘}{H}}_{12}\right]}{\left[H_{12}-\frac{1}{2}\left(H_{1}+H_{2}\right)\right]H_{12}}\right],$$ (16)

where

$$\text{Var}\left[{\overset{˘}{H}}_{12}-\frac{1}{2}\left({\overset{˘}{H}}_{1}+{\overset{˘}{H}}_{2}\right)\right]=\text{Var}\left[{\overset{˘}{H}}_{12}\right]+\frac{1}{4}\text{Var}\left[{\overset{˘}{H}}_{1}\right]+\frac{1}{4}\text{Var}\left[{\overset{˘}{H}}_{2}\right]-\left(\text{Cov}\left[{\overset{˘}{H}}_{12},{\overset{˘}{H}}_{1}\right]+\text{Cov}\left[{\overset{˘}{H}}_{12},{\overset{˘}{H}}_{2}\right]\right).$$ (17)

In the Appendix, we provide a derivation of the variance and covariance components of Equations 16 and 17. For each of these equations, the result and proof are fairly long, and do not simplify when arranged into Equation 16.

### Data availability

The authors state that all data necessary for confirming the conclusions presented in the article are represented fully within the article.

## Results

### Analytical validation of ${\tilde{\text{H}}}_{\text{BLUE}}$

We tested the performance of ${\tilde{H}}_{\text{BLUE}}$ using both theory and simulations against that of the unbiased estimator $\tilde{H}$ (DeGiorgio *et al.* 2010), and of $\hat{H}$ (Nei and Roychoudhury 1974). Here, we applied the estimators to samples of individuals wherein each individual was related to exactly one other. Thus, for samples of size *n* individuals, the number of relative pairs was *n*/2. When inbred or closely related individuals are included in a sample, $\hat{H}$ is a biased estimator of gene diversity for which we use the symbol ${\hat{H}}_{\text{full}}.$ To construct an unbiased estimator with $\hat{H},$ we also applied $\hat{H}$ to a reduced sample in which one member of each relative pair was removed randomly for samples containing only diploid individuals, and the haploid member was removed for each haploid-diploid (*i.e.*, male-female) pair (reduced sample size of *n*/2), and we denote this estimator by ${\hat{H}}_{\text{red}}.$ To evaluate the performance of the four estimators (${\hat{H}}_{\text{full}},$ ${\hat{H}}_{\text{red}},$ $\tilde{H},$ and ${\tilde{H}}_{\text{BLUE}}$), we modified the factors upon which their variance depends: true locus expected heterozygosity (*H*), sample size *n*, and relatedness of individuals within the sample (Φ).

### Effect of true locus expected heterozygosity, H, on estimators

**Figure 1:**
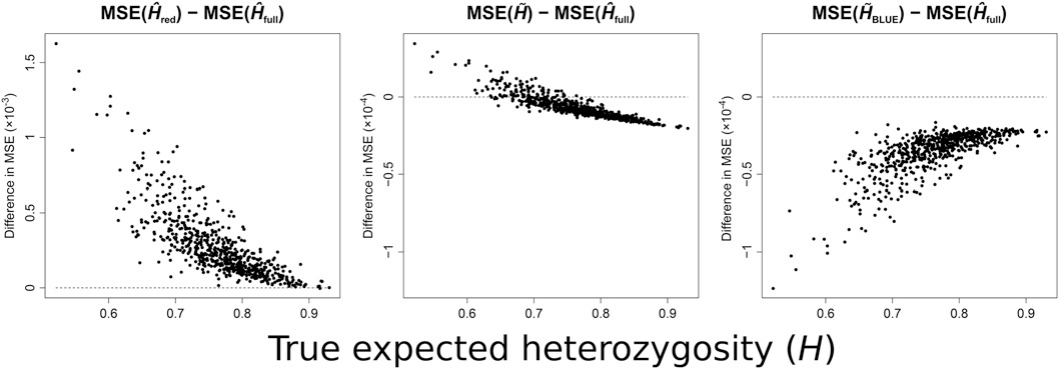
Theoretical difference in MSE between the unbiased estimator ${\hat{H}}_{\text{red}}$ (left), $\tilde{H}$ (center), or ${\tilde{H}}_{\text{BLUE}}$ (right), and the biased estimator ${\hat{H}}_{\text{full}}$ calculated at each of 645 microsatellite loci (0.5212≤H≤0.9301) in the MS5795 dataset for samples of 60 diploid individuals containing some inbred relative pairs. Each sampled individual was related to exactly one other, and samples contained 10 pairs of inbred full-siblings (Φ=3/8), 10 pairs of outbred full-siblings (Φ=1/4), and 10 outbred avuncular pairs (Φ=1/8). Dotted lines in each plot correspond to a difference in MSE of zero with ${\hat{H}}_{\text{full}}.$ See File S1 for the true expected heterozygosity values incorporated into analytical calculations.

We first evaluated the theoretical bias, variance, and mean squared error (MSE) of each estimator across the 645 human microsatellite loci from across the genome in the composite dataset MS5795 of Pemberton *et al.* (2013), where MSE is the sum of the squared bias and variance. The data used in our analyses is freely available online within File S1 of Pemberton *et al.* (2013) (http://www.g3journal.org/content/early/2013/03/27/g3.113.005728/suppl/DC1). We took the sample allele frequencies calculated from all individuals in the MS5795 dataset as the true population allele frequencies for the variance calculations, and, from these, determined the true expected heterozygosity at each locus using Equation 1 (see File S1; incorporated into Equation A10). Here, each sample contained 60 diploid individuals composed of 10 inbred full-sibling, 10 outbred full-sibling, and 10 outbred avuncular pairs. Each point in Figure 1 and Figure S1 represents a single analytical computation for a sample of 60 (or 30 for ${\hat{H}}_{\text{red}}$) individuals at a microsatellite locus. We report the approximate variance and MSE because each individual is related to exactly one other in the sample, satisfying the assumption of Equation 11. Further, under this scenario DeGiorgio *et al.* (2010) showed that this was a reasonable approximation of the exact variance.

We begin by demonstrating the relative performance of the unbiased estimators ${\hat{H}}_{\text{red}},$ $\tilde{H},$ and ${\tilde{H}}_{\text{BLUE}},$ measured in terms of MSE, against the biased estimator ${\hat{H}}_{\text{full}}$ (Figure 1). While the variance of ${\hat{H}}_{\text{full}}$ is invariably smaller than that of the other estimators, and the MSE and variance of each estimator decrease with increasing locus expected heterozygosity (0.5212≤H≤0.9301), ${\hat{H}}_{\text{full}}$ accumulates bias quadratically with increasing *H*, and thus yields an increasingly unreliable estimate with increasing site diversity (Figure S1A, left). However, the effect of this trend differs for each comparison. The MSE of ${\hat{H}}_{\text{red}}$ always exceeds that of ${\hat{H}}_{\text{full}},$ because the removal of relatives to create the reduced sample causes a substantial increase in estimator variance, though, for high diversity markers, the MSE values of ${\hat{H}}_{\text{full}}$ and ${\hat{H}}_{\text{red}}$ converge (Figure 1, left). In contrast, $\tilde{H}$ outperforms ${\hat{H}}_{\text{full}}$ for most loci, demonstrating that the rate of decrease in MSE with increasing *H* is greater for $\tilde{H}$ than for ${\hat{H}}_{\text{full}}$ (Figure 1, center). Interestingly, the comparison of ${\tilde{H}}_{\text{BLUE}}$ with ${\hat{H}}_{\text{full}}$ shows an opposite trend to the preceding two. Despite the impact of bias, the decrease in variance of ${\hat{H}}_{\text{full}}$ over the analyzed range outpaces that of ${\tilde{H}}_{\text{BLUE}}.$ Even so, ${\tilde{H}}_{\text{BLUE}}$ uniformly yields a smaller MSE for the analyzed diploid samples (which contain a proportion of inbred individuals) across all loci (Figure 1, right).

To validate these theoretical predictions, we simulated 30 independent genotypes for each locus, and, for each independent genotype, simulated a single relative’s genotype (inbred full-sibling, outbred full-sibling, or avuncular). Briefly, we generated the independent genotypes by sampling alleles uniformly at random from the distribution of allele frequencies at each microsatellite locus, and generated relatives by copying zero, one, or two alleles from the relative according to the probability the pair would share zero, one, or two alleles IBD [see Lange (2002), Chapter 5]. The patterns observed for the simulated data accord with those of the theoretical predictions (Figure S2, each point is based on $10^{4}$ simulations). It is clear from these results that locus expected heterozygosity is heavily influential on estimator MSE. However, we also find that the observed value of expected heterozygosity for a locus normalized to its range of expected heterozygosity values has an impact on estimator MSE. The maximum and minimum values of expected heterozygosity for a locus depend on the number of distinct alleles (*I*), and the frequency of the most frequent allele (*M*), at that locus [see Theorem 2 of Reddy and Rosenberg (2012)]. We quantify proximity of *H* for a locus to its maximum possible value as B=D/R, where *D* is the observed value of expected heterozygosity for a locus minus its minimum possible value given *I* and *M*, and *R* is the maximum minus the minimum value of expected heterozygosity, given *I* and *M*, such that B∈[0,1]. Loci with a smaller value of *B* yield a smaller MSE for all estimators (Figure S3).

### Effect of sample size, n, on estimators

**Figure 2:**
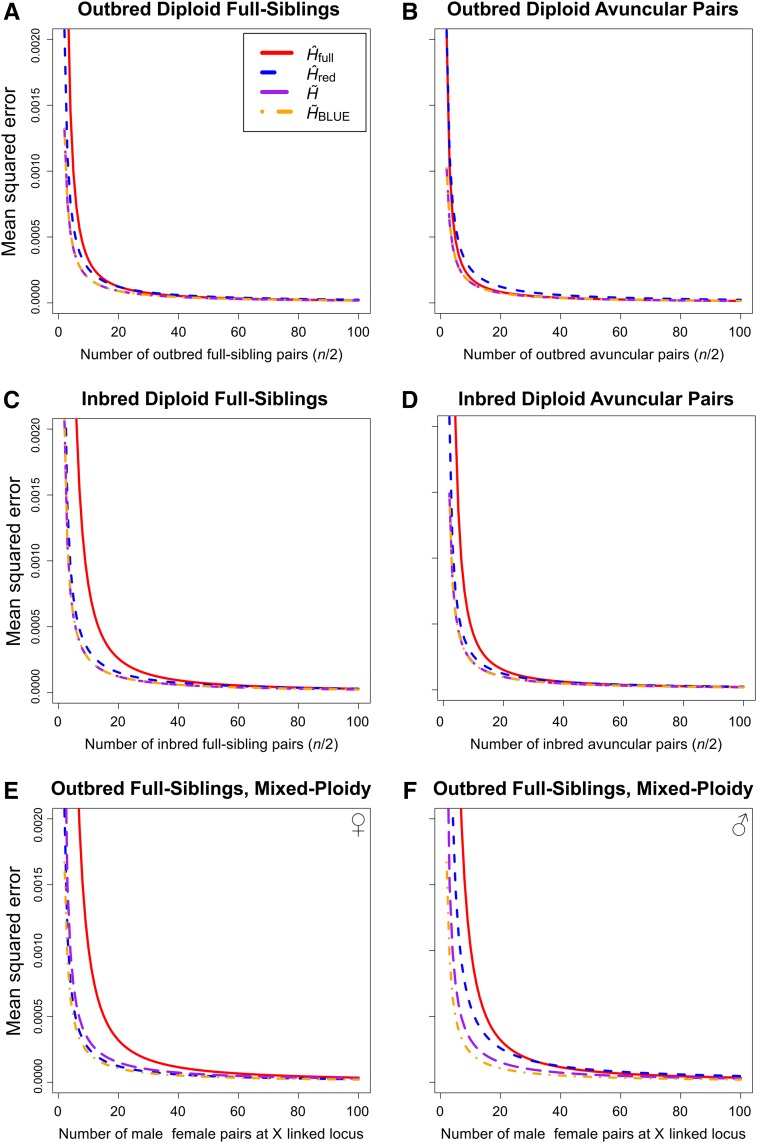
Theoretical MSE as a function of sample size for samples of outbred diploid full-siblings (A), outbred diploid avuncular pairs (B), inbred diploid full-siblings (C), inbred diploid avuncular pairs (D), male-female full siblings at an X-linked locus with the reduced set omitting males and retaining females (E), and male-female full siblings at an X-linked locus with the reduced set omitting females and retaining males (F). The samples were evaluated for the D3S2427 locus (H=0.9301), and sample size was always twice the number of relative pairs included in the sample for samples containing 2–100 relative pairs. Each individual in the sample was related to exactly one other.

We next examined the properties of each estimator as a function of sample size. All estimators perform increasingly well for samples of increasing size. We demonstrate this property by measuring estimator MSE for samples containing 2–100 relative pairs of various type and ploidy at the D3S2427 locus, selected to highlight the improved performance of ${\tilde{H}}_{\text{BLUE}}$ as the bias of ${\hat{H}}_{\text{full}}$ increases (H=0.9301; Figure 2). For these tests, we considered only a single relative pair type at a time. The unbiased estimators $\tilde{H}$ and ${\tilde{H}}_{\text{BLUE}}$ perform identically for diploid samples of first- and second-degree relative pairs regardless of inbreeding (Figure 2, A–D). Additionally, estimator MSE is uniformly smaller for samples containing only second-degree relative pairs than it is for samples containing only first-degree pairs (*cf*. Figure 2, A and B, and Figure 2, C and D; see also, Figure S4A). However, ${\tilde{H}}_{\text{BLUE}}$ unambiguously outperforms the other estimators with relative pairs of varying ploidy (in this case, male-female full-sibling pairs at an X-linked locus). In this scenario, ${\hat{H}}_{\text{red}}$ provides a more accurate estimate of expected heterozygosity than does $\tilde{H}$ when the reduced set is created by removing only males from the original while retaining females (Figure 2E). When all females are removed instead, and males retained (Figure 2F), the MSE of ${\hat{H}}_{\text{red}}$ is markedly the largest of the four estimators because 2/3 of the alleles in the sample are discarded, rather than 1/3. For samples with inbred full-siblings whose parents are brother and sister (Figure 2, C and D), the trend of MSE with sample size mirrors that of outbred diploid samples (Figure 2, A and B), but with larger MSE. However, the relative performance of ${\hat{H}}_{\text{full}}$ is notably worse for samples containing inbred diploid avuncular pairs (Figure 2D) than for samples containing outbred diploid avuncular pairs (Figure 2B). That is, its MSE remains greater than, or equal to, that of the other estimators over the range of sample sizes considered for the inbred diploid avuncular pair scenario (Figure 2D), but consistently has smaller MSE than ${\hat{H}}_{\text{red}}$ for the outbred diploid avuncular pair scenario (Figure 2B). Generally, increasing the sample size is most effective for samples of <20 individuals, and it is over this range that the difference in performance of the estimators is most apparent.

### Effect of varying sample relative pair composition on estimators

**Figure 3:**
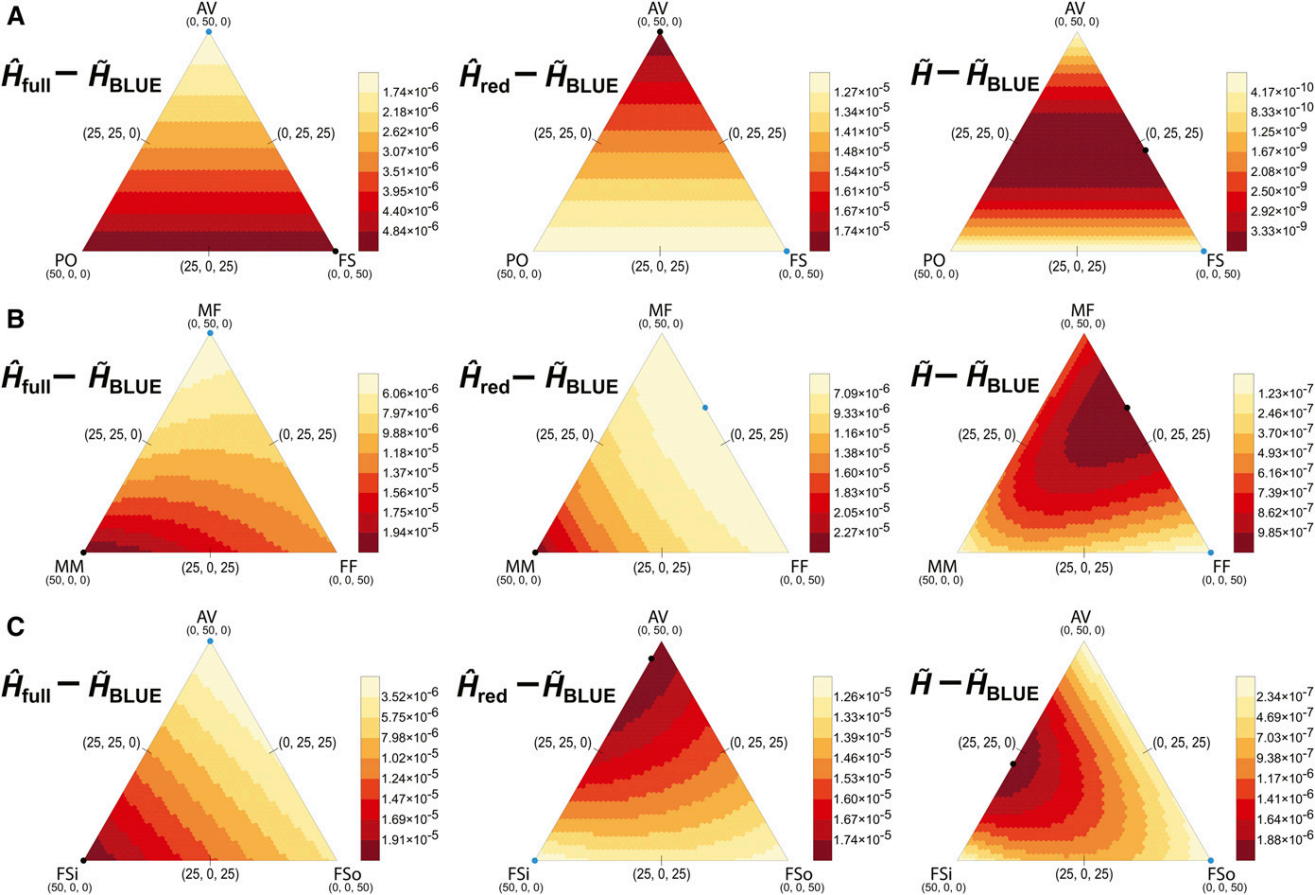
Theoretical difference in MSE between ${\hat{H}}_{\text{full}}$ (left), ${\hat{H}}_{\text{red}}$ (center), or $\tilde{H}$ (right), and ${\tilde{H}}_{\text{BLUE}},$ for samples of 100 (A) outbred diploid individuals, (B) male and female individuals at an X-linked locus, or (C) diploid individuals wherein some full siblings are inbred with brother-sister parents. The samples and MSE values considered for each subtraction were modeled on the D3S2427 locus (H=0.9301). Each sample contained 50 relative pairs, such that each individual was related to exactly one other. Each sample configuration is a single point in the space of a heat map defined by three coordinates (each representing the count of a relative pair type). For each configuration, the MSE of ${\tilde{H}}_{\text{BLUE}}$ is subtracted from that of the other estimators, yielding a value >0. Samples were composed of one to three relative pair types where the vertex of each heat map represents a sample with only a single relative pair type. The relative pair types were (A) parent-offspring (PO), second-degree avuncular (AV), and full-sibling (FS), (B) male-male (MM), male-female (MF), and female-female (FF) full-sibling such that the number of males and females in each sample is not fixed, or (C) inbred full-sibling (FSi), second-degree avuncular (AV), and outbred full-sibling (FSo). Blue and black points indicate the smallest and largest values, respectively, on each map. Threshold values for coloration are indicated in the scales to the right of each heat map, with smaller values colored lighter. Note that the scales are not identical across heat maps. The values upon which these subtractions are based are represented as heat maps in (A) Figure S4A, (B) Figure S4B, or (C) Figure S4C.

Finally, we calculated the MSE of each estimator for all 1326 combinations of one to three relative pair types for samples of 100 individuals fixed at 50 relative pairs, which we represent as triangular heat maps, across samples containing outbred diploids, males and females at an X-linked locus, or inbred diploids (each individual related to exactly one other; Figure 3, Figure S4, Figure S5, Figure S6, Figure S7, and Figure S8). The kinship coefficients (Φ) for each relative pair type considered across our tests are defined in Lange (2002, Chapter 5) and DeGiorgio *et al.* (2010, see Table 2), and modeled on the D3S2427 locus (H=0.9301).

The outbred diploid samples included parent-offspring (Φ=1/4), avuncular (Φ=1/8), and full-sibling (Φ=1/4) relative pairs. Because parent-offspring and full-sibling pairs have the same kinship coefficient, the heat maps in Figure 3, Figure S4A, Figure S5A, Figure S6A, and Figure S7A are symmetrical with parent-offspring and full-sibling pairs on the bottom vertices, and avuncular pairs on the top vertex. ${\hat{H}}_{\text{red}}$ yielded the largest MSE of the four estimators, and this value was constant throughout the space of the heat map (Figure S4A, second triangle), because all reduced sets are identical for outbred diploid samples. ${\tilde{H}}_{\text{BLUE}}$ consistently yielded the smallest MSE across configurations (Figure S4A, fourth triangle). As was the case in Figure 2, the MSE of the estimators ${\hat{H}}_{\text{full}},$ $\tilde{H},$ and ${\tilde{H}}_{\text{BLUE}}$ was smallest for samples with only avuncular pairs, because these contain fewer dependent allele observations on average. We observed these features in simulated data as well (Figure S8A).

Although ${\tilde{H}}_{\text{BLUE}}$ performed best overall for samples including outbred diploid relative pairs at D3S2427, the estimator with the smallest variance in all situations is the biased estimator ${\hat{H}}_{\text{full}}$ (Figure S6A). However, because its squared bias increases with the number of first-degree pairs (Figure S5A), its relative performance declines compared to ${\tilde{H}}_{\text{BLUE}}$ as more of these pairs are sampled (Figure 3A, left triangle). The relative performance of ${\hat{H}}_{\text{red}}$ is highest when the number of first degree pairs is maximized, but this is due to the decreasing performance of ${\tilde{H}}_{\text{BLUE}}$ as more dependent observations are included (Figure 3A, center triangle). While the difference in MSE between $\tilde{H}$ and ${\tilde{H}}_{\text{BLUE}}$ is always slight for samples of noninbred diploids, these values diverge as the complexity of the sample increases (Figure 3A, right triangle). That is, as the numbers of first- and second-degree pairs approach each other, ${\tilde{H}}_{\text{BLUE}}$ emerges decisively as the more accurate estimator, with the maximum value of this difference reached at 23 second-degree and 27 first-degree pairs. Thus, while the performance of the estimators for a sample containing relatives follows the same general trend, ${\tilde{H}}_{\text{BLUE}}$ provides the greatest accuracy for heterogeneous samples of outbred diploid individuals.

We also considered the relative performance of each estimator when using either the BLUE (${\tilde{p}}_{i}$) or the sample proportion (${\hat{p}}_{i}$) to estimate allele frequencies. Notably, all estimators perform best when the BLUE (${\tilde{p}}_{i}$) of allele frequency rather than the sample proportion (${\hat{p}}_{i}$) is used to infer population allele frequencies. We calculated the theoretical MSE for each estimator once with ${\hat{p}}_{i},$ and once with ${\tilde{p}}_{i},$ across all combinations of relative pairs for diploid individuals at the D3S2427 locus and mapped its value for the estimate with ${\hat{p}}_{i}$ minus the estimate with ${\tilde{p}}_{i}$ (Figure S7A). Because both frequency estimations yield the same values in samples of unrelated individuals, ${\hat{H}}_{\text{red}}$ performs identically for ${\hat{p}}_{i}$ and ${\tilde{p}}_{i},$ and is not included. The MSE of an estimator calculated with ${\tilde{p}}_{i}$ is always smaller than that of the estimator calculated with ${\hat{p}}_{i},$ and the pattern of divergence between their MSEs follows a similar trend across all estimators, resembling the rightmost panel in Figure 3A. This result suggests that the difference in MSE between $\tilde{H}$ and ${\tilde{H}}_{\text{BLUE}}$ is driven primarily by the difference in performance between ${\hat{p}}_{i}$ and ${\tilde{p}}_{i}.$ Both the ${\hat{p}}_{i}$ and ${\tilde{p}}_{i}$ estimators yield the same value at the vertices of the triangles, and the difference in their MSEs reaches a maximum at 22 second-degree pairs for ${\hat{H}}_{\text{full}}$ and 24 second-degree pairs for $\tilde{H}$ and ${\tilde{H}}_{\text{BLUE}}$ (Figure S7A, center and right triangles). The MSE of ${\tilde{H}}_{\text{BLUE}}$ calculated with ${\hat{p}}_{i}$ is, at most, on the order of $10^{-9}$ greater than that of ${\tilde{H}}_{\text{BLUE}}$ calculated with ${\tilde{p}}_{i},$ indicating its robustness to variance in allele frequency determination (Figure S7A, right triangle). In contrast, the other estimators return a maximum difference in MSE on the order of $10^{-7}.$ The estimation of expected heterozygosity with ${\hat{H}}_{\text{full}},$ $\tilde{H},$ or ${\tilde{H}}_{\text{BLUE}}$ will always yield a smaller MSE for samples of outbred, diploid individuals when ${\tilde{p}}_{i}$ rather than ${\hat{p}}_{i}$ is taken as the estimator of population allele frequency.

We repeated these tests in samples of mixed ploidy (Figure 3B, Figure S4B, Figure S5B, Figure S6B, Figure S7B, and Figure S8B), and ${\tilde{H}}_{\text{BLUE}}$ emerged similarly superior to the other estimators, once again yielding the smallest MSE. We analyzed the D3S2427 locus as X-linked for these tests, counting males as haploid and females as diploid, and observed full-sibling pairs [similarly to DeGiorgio *et al.* (2010), Φ=1/2 for male-male pairs, Φ=1/4 for male-female pairs, and Φ=3/8 for female-female pairs] for samples of 100 individuals and 50 relative pairs. All estimators reach their maximum MSE in samples containing only male-male pairs (Figure S4B). This is because the number of independent observations (indicated by a larger mean kinship coefficient) is smallest when there are no females in the sample. Correspondingly, the estimators yield smaller MSE values with increasing incorporation of male-female pairs. The minimum MSE of ${\hat{H}}_{\text{full}}$ is reached at 50 male-female pairs, as with $\tilde{H}$ and ${\tilde{H}}_{\text{BLUE}}$ because its squared bias (Figure S5B) decreases with increasing male-female pairs, though its variance is smallest at 50 female-female pairs, due to the greater number of alleles in the sample (Figure S6B). To create the reduced sets, males were removed from male-female pairs to minimize the subsequent increase in MSE. That is, the removal of males removes 1/3 of the allele copies from the sample, rather than 2/3 if females are removed, or 1/2 for a pair of same-ploidy individuals, and so ${\hat{H}}_{\text{red}}$ has the same value across samples with the same number of male-male pairs (Figure S4B, second triangle).

The direct comparison of ${\tilde{H}}_{\text{BLUE}}$ with the other estimators once again yielded different signatures for each subtraction for mixed-ploidy samples (Figure 3B). The point of greatest difference in MSE between ${\hat{H}}_{\text{full}}$ and ${\tilde{H}}_{\text{BLUE}}$ occurs when all relative pairs are male-male, while the point of least difference occurs for samples of only male-female pairs (Figure 3B, left triangle). This pattern broadly resembles the squared bias of ${\hat{H}}_{\text{full}}$ (Figure S5B, first triangle), underscoring the effect of bias on estimator performance. The pattern of difference in performance between ${\hat{H}}_{\text{red}}$ and ${\tilde{H}}_{\text{BLUE}}$ differs markedly, and the two estimators perform most similarly as the number of male-male pairs decreases, reaching a minimum at 33 male-female pairs plus 17 female-female pairs (Figure 3B, middle triangle). $\tilde{H}$ yields the closest MSE to that of ${\tilde{H}}_{\text{BLUE}}$ for all relative pair configurations, and their difference is, at most, on the order of $10^{-6}$ (Figure 3B, right triangle). The pattern here mainly reflects the difference in performance between ${\hat{p}}_{i}$ and ${\tilde{p}}_{i}$ estimates of population allele frequency, as in Figure S7B, where ${\tilde{p}}_{i}$ estimators yield increasingly smaller comparative MSE values as the numbers of relative pairs in the sample approach each other.

We repeated the preceding tests once more for a sample in which full-siblings resulting from a brother-sister mating were included alongside second-degree and outbred full-sibling pairs (Figure 3C, Figure S4C, Figure S5C, Figure S6C, Figure S7C, and Figure S8C). Here, the kinship of inbred individuals with each other was 3/8 rather than 1/4. For all estimators, the inclusion of inbred full-siblings increased the MSE of the estimator, with a maximum MSE at 50 inbred full-sibling pairs, and a minimum at 50 second-degree pairs. For ${\hat{H}}_{\text{red}},$ this minimum was also reached for any sample in which there were no inbred individuals, because the reduced sample is identical for these (Figure S4C, second triangle). Again, ${\tilde{H}}_{\text{BLUE}}$ was the least errant estimator across the space of sample configurations (Figure S4C, fourth triangle), and its advantage over the other estimators differs for each estimator (Figure 3C). Because the bias of ${\hat{H}}_{\text{full}}$ is largest at 50 inbred full-sibling pairs, the greatest difference in performance between it and ${\tilde{H}}_{\text{BLUE}}$ is at this point (Figure 3C, left triangle). Meanwhile, the largest differences in MSE between ${\hat{H}}_{\text{red}}$ and ${\tilde{H}}_{\text{BLUE}}$ are near the top vertex, where second-degree relative pairs predominate, while the smallest are toward the bottom vertices (Figure 3C, center triangle). The difference in MSE between $\tilde{H}$ and ${\tilde{H}}_{\text{BLUE}}$ is at least an order of magnitude less than for the other comparisons, and increases for increasing sample complexity, but reaches its maximum for samples of 28 inbred full-sibling plus 22 second-degree pairs (Figure 3C, right triangle). This pattern reflects the decreased MSE for the estimators when calculated with ${\tilde{p}}_{i}$ compared to their calculation with ${\hat{p}}_{i}$ (Figure S7C). Ultimately, the performance of the estimators of expected heterozygosity across varying sample compositions depends on the estimator of allele frequency incorporated into the expected heterozygosity calculation. No matter the sample type, estimators based on ${\tilde{p}}_{i}$ outperform estimators based on ${\hat{p}}_{i},$ and ${\tilde{H}}_{\text{BLUE}}$ outperforms ${\hat{H}}_{\text{full}},$ ${\hat{H}}_{\text{red}},$ and $\tilde{H}.$

### Tests of ${\tilde{\text{H}}}_{\text{BLUE}}$ on single-nucleotide polymorphism (SNP) loci

Because SNP datasets are more common in recent studies, we performed analyses equivalent to our microsatellite analyses for 50 hypothetical SNP loci. These loci were biallelic with minor allele frequency (MAF) between 0.01 and 0.5, with increments of 0.01, corresponding to expected heterozygosity values ranging from 0.0198 to 0.5. We first measured the difference in MSE of ${\hat{H}}_{\text{full}}$ with that of ${\hat{H}}_{\text{red}},$ $\tilde{H},$ or ${\tilde{H}}_{\text{BLUE}}$ as a function of true locus expected heterozygosity (*H*), as we did in Figure 1 (Figure S9). For each locus, the MSE of ${\tilde{H}}_{\text{BLUE}}$ was smallest, while that of ${\hat{H}}_{\text{full}}$ was generally second-smallest, following the trend for microsatellite loci visible in Figure 1, wherein less diverse loci yielded a smaller MSE for ${\hat{H}}_{\text{full}}$ than for $\tilde{H}.$ However, unlike for microsatellite loci, estimator MSE peaks midway through the range of evaluated SNP loci, such that the smallest MSE values lie at either extreme of the range and the largest MSE value, as well as the largest difference in MSE values for all comparisons, is at the locus with MAF=0.15 (H=0.255). Additionally, ${\hat{H}}_{\text{full}}$ performs comparatively better than ${\hat{H}}_{\text{red}}$ (Figure S9, left) and $\tilde{H}$ (Figure S9, center) as *H* approaches 0.255, but is outperformed by these unbiased estimators as *H* approaches 0.5. Once more, the trend is opposite for the comparison between ${\hat{H}}_{\text{full}}$ and ${\tilde{H}}_{\text{BLUE}},$ showing the greatest comparative performance by ${\tilde{H}}_{\text{BLUE}}$ at the same locus (MAF =0.15, H=0.255). Thus, considering the results presented in Figure 1 and Figure S9, the greatest relative performance of ${\tilde{H}}_{\text{BLUE}}$ for inbred samples is achieved at loci for which estimator MSE is largest.

We next examined the effect of sample size on estimator performance for hypothetical samples of outbred diploid, inbred diploid, and outbred male-female relative pairs at the simulated locus with MAF =0.05 (H=0.095). As we varied the sample size from two relative pairs to 100 (each individual related to exactly one other, one relative pair type per sample), we found that ${\tilde{H}}_{\text{BLUE}}$ yielded the smallest MSE of all estimators only for samples containing male-female full-sibling pairs modeled at an X-linked locus (Figure S10, E and F). This observation mirrors the trend seen in Figure 2, wherein ${\tilde{H}}_{\text{BLUE}}$ outperformed the other estimators across all sample sizes. However, ${\hat{H}}_{\text{full}}$ yielded the smallest MSE across all sample sizes for outbred and inbred diploid full-siblings and avuncular pairs (Figure S10, A–D). This result is because the samples modeled here are minimally complex, with only one relative pair type, and modeled for a highly homozygous marker—two conditions under which the low bias and variance of ${\hat{H}}_{\text{full}}$ result in favorable performance.

Finally, we analyzed estimator performance once more for the locus with MAF =0.05 (H=0.095), for a sample of 50 individuals across changing outbred diploid, inbred diploid, and male-female full-sibling relative pair compositions (Figure S11, A–C). We display these results as heat maps, and find that our results here are broadly concordant with those for the D3S2427 human microsatellite locus (H=0.9301). As with the experiments displayed in Figure S10, the least complex samples yielded a smaller MSE for ${\hat{H}}_{\text{full}}$ estimates than for ${\tilde{H}}_{\text{BLUE}}$ estimates. Correspondingly, samples whose relative pair compositions resulted in fewer independent allele observations were more accurately and precisely evaluated with ${\tilde{H}}_{\text{BLUE}}.$ Thus, while sampling lower-diversity markers may occasionally favor the use of ${\hat{H}}_{\text{full}},$ the inclusion of two or more relative pair types in the sample is likely to bias ${\hat{H}}_{\text{full}}$, and require the use of ${\tilde{H}}_{\text{BLUE}}$ to yield accurate inferences.

### Empirical application of ${\tilde{\text{H}}}_{\text{BLUE}}$

To conclude our investigation into the performance of ${\tilde{H}}_{\text{BLUE}},$ we applied it to empirical data from the MS5795 dataset. We retrieved human microsatellite data from 5795 individuals (11,590 allele copies) across 645 autosomal loci sampled genome wide. We assumed the mean value across loci for ${\hat{H}}_{\text{red}}$ in each of 267 populations to be the true expected heterozygosity value for these populations, as it is an unbiased estimate. We additionally chose to compare the other estimators with ${\hat{H}}_{\text{red}},$ because an important basis for their evaluation is their agreement with this unbiased estimator, irrespective of the data to which they are applied.

To emphasize this, we performed three Wilcoxon signed-rank tests to compare the ranking of populations by their mean expected heterozygosity across all loci calculated with ${\hat{H}}_{\text{red}},$ and either ${\hat{H}}_{\text{full}},$ $\tilde{H},$ or ${\tilde{H}}_{\text{BLUE}}$ (Table 1). At the α<0.01 significance level, the comparisons showed that the inclusion of relatives for ${\hat{H}}_{\text{full}}$ was highly significant on the rankings it yielded, indicating that not correcting for relatedness among samples can significantly alter the estimates of expected heterozygosity. However, both $\tilde{H}$ and, especially, ${\tilde{H}}_{\text{BLUE}},$ yielded *P*-values greater than the threshold for the test against ${\hat{H}}_{\text{red}}.$ These results indicate that the estimates of expected heterozygosity are not significantly affected by the inclusion of related individuals in the sample when relatedness is taken into account. Furthermore, a test between $\tilde{H}$ and ${\tilde{H}}_{\text{BLUE}}$ yielded a *P*-value of $3.44\times 10^{-2},$ suggesting no significant difference in the ranking of populations by mean expected heterozygosity with these two estimators.

**Table 1:** Wilcoxon signed-rank test for mean across loci of ${\hat{H}}_{\text{red}}$ with ${\hat{H}}_{\text{full}},$ $\tilde{H},$ and ${\tilde{H}}_{\text{BLUE}}$ for the 93 populations whose samples contained related individuals

Comparison	*P*-Value for Wilcoxon Signed-Rank Test
${\hat{H}}_{\text{red}}$ with ${\hat{H}}_{\text{full}}$	$4.39\times 10^{-15}$
${\hat{H}}_{\text{red}}$ with $\tilde{H}$	$1.00\times 10^{-2}$
${\hat{H}}_{\text{red}}$ with ${\tilde{H}}_{\text{BLUE}}$	0.255

**Figure 4:**
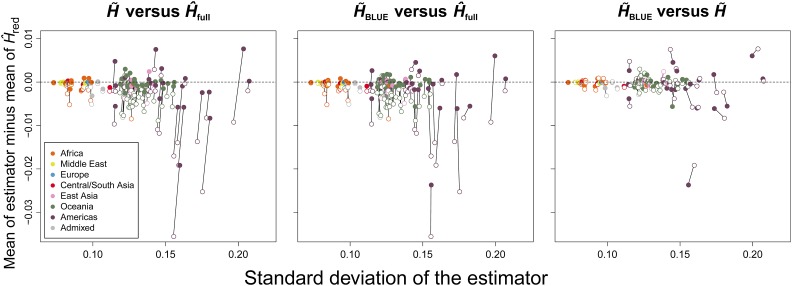
Application of the estimators to dataset MS5795. Here, we show a comparison of two estimators at a time (${\hat{H}}_{\text{full}},$ $\tilde{H},$ or ${\tilde{H}}_{\text{BLUE}}$) by the difference in their mean with that of ${\hat{H}}_{\text{red}}$ across the 645 sampled microsatellite loci of MS5795 (vertical axis), and by their SDs (horizontal axis). The horizontal dotted line corresponds to no difference between the mean of the estimator and the mean of the unbiased estimator ${\hat{H}}_{\text{red}}.$ Solid lines connect calculations made for the same population with different estimators. Points are colored by geographic division defined in the dataset. Only the 93 populations with relatives in their samples were included because ${\hat{H}}_{\text{full}},$ $\tilde{H},$ and ${\tilde{H}}_{\text{BLUE}}$ return the same value for samples of unrelated individuals. In the leftmost plot, open points are estimates for ${\hat{H}}_{\text{full}},$ while closed points are for $\tilde{H}.$ In the center plot, open points are estimates for ${\hat{H}}_{\text{full}},$ while closed points are for ${\tilde{H}}_{\text{BLUE}}.$ In the rightmost plot, open points are estimates for $\tilde{H},$ while closed points are for ${\tilde{H}}_{\text{BLUE}}.$

Although the unbiased estimators $\tilde{H}$ and ${\tilde{H}}_{\text{BLUE}}$ have smaller MSE than ${\hat{H}}_{\text{full}}$ for samples with related individuals, their variance tends to be larger than that of ${\hat{H}}_{\text{full}}.$ DeGiorgio *et al.* (2010) previously showed that the difference in SD of $\tilde{H}$ with ${\hat{H}}_{\text{full}}$ was small, while the mean values of $\tilde{H}$ and ${\hat{H}}_{\text{red}}$ were much more similar to each other than either of them was to the mean of ${\hat{H}}_{\text{full}}.$ We again show this to be the case, and find as well that ${\tilde{H}}_{\text{BLUE}}$ not only repeats, or improves upon, the concordance of $\tilde{H}$ with ${\hat{H}}_{\text{red}},$ but, in some cases, ${\tilde{H}}_{\text{BLUE}}$ has a smaller SD than does ${\hat{H}}_{\text{full}}$ (Figure 4, left and center panels). A direct comparison of the performance of $\tilde{H}$ against that of ${\tilde{H}}_{\text{BLUE}}$ (Figure 4, right panel) shows that ${\tilde{H}}_{\text{BLUE}}$ has a generally improved SD, and similarity to the ${\hat{H}}_{\text{red}}$ estimate over $\tilde{H}.$ For some samples (primarily those from the Americas), this is not the case, possibly because all close relatives were not identified in the original dataset, resulting in an incorrect kinship matrix for calculation of the statistic.

### Improving estimates of ${\text{F}}_{\text{ST}}$ by application of ${\tilde{\text{H}}}_{\text{BLUE}}$

**Figure 5:**
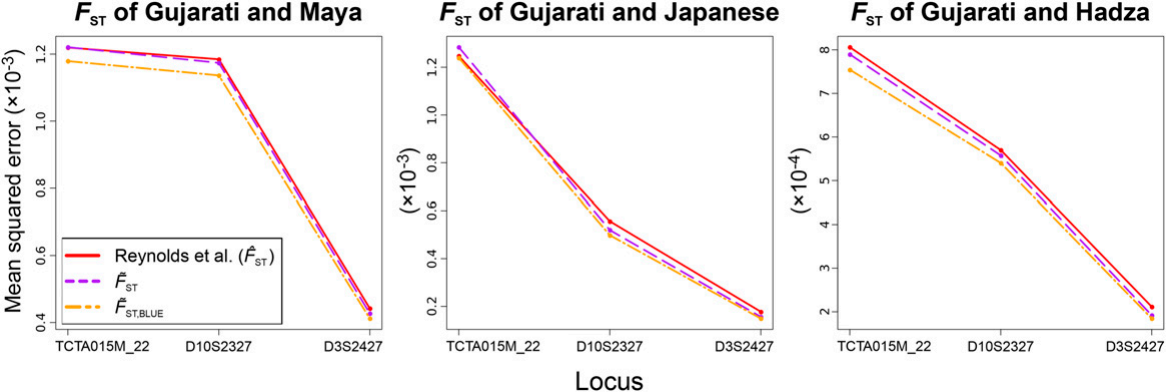
Application of the estimators ${\hat{H}}_{\text{full}},$ $\tilde{H},$ and ${\tilde{H}}_{\text{BLUE}}$ to the calculation of $F_{\text{ST}}$ as ${\hat{F}}_{\text{ST}},$ ${\tilde{F}}_{\text{ST}},$ and ${\tilde{F}}_{\text{ST},\text{BLUE}},$ respectively, using simulated data for the Gujarati sample, with either the Maya (left), Japanese (center), or Hadza (right) samples, showing MSE on the vertical axis. The Reynolds *et al.* (1983) estimator is equivalent to the application of ${\hat{H}}_{\text{full}}$ in calculating population differentiation. The simulated samples contained 60 individuals and 30 relative pairs, of which 10 were inbred full-siblings, 10 were outbred full-siblings, and 10 were outbred avuncular pairs. Each individual was related to exactly one other, and the data were simulated following the same probabilistic method as employed to generate Figure S2. The three loci displayed on the horizontal axis are the least diverse, median diverse, and most diverse loci of the 645 MS5795 human microsatellites.

We predicted that the smaller MSE of ${\tilde{H}}_{\text{BLUE}}$ would translate to improved accuracy for estimators that are summaries of expected heterozygosity when samples contain related individuals. To test this hypothesis, we calculated the population differentiation statistic, $F_{\text{ST}}$ (Equation 4), for pairs of populations whose samples in the MS5795 dataset contained related individuals. Our intent was to compare the MSE and bias of the commonly used $F_{\text{ST}}$ estimator of Reynolds *et al.* (1983), which is based on ${\hat{H}}_{\text{full}},$ and which we label as ${\hat{F}}_{\text{ST}},$ to an estimate of $F_{\text{ST}}$ calculated from ${\tilde{H}}_{\text{BLUE}},$ which we label ${\tilde{F}}_{\text{ST},\text{BLUE}}.$ The formulas for these estimators follow the form of the general estimator of $F_{\text{ST}}$ (Equation 14). We first measured the MSE of both methods (and an estimate using $\tilde{H},$ ${\tilde{F}}_{\text{ST}}$) on simulated data, where the $F_{\text{ST}}$ of pairs of populations with samples of size 60 diploids each (30 relative pairs, 10 inbred full-sibling, 10 outbred full-sibling, and 10 avuncular pairs; Figure 5) was averaged across $10^{4}$ simulated replicates. The calculations included here were performed for simulated Gujarati and Maya (left), Gujarati and Japanese (center), or Gujarati and Hadza (right) samples for the least diverse (TCTA015M_22), median diverse (D10S2327), and most diverse (D3S2427) loci of the MS5795 dataset, following their allele frequency distribution in MS5795. ${\tilde{F}}_{\text{ST},\text{BLUE}}$ consistently has a smaller MSE than the others, and the MSE of all estimators of $F_{\text{ST}}$ decreases with increasing locus diversity, as the MSE of the estimator of expected heterozygosity decreases.

**Figure 6:**
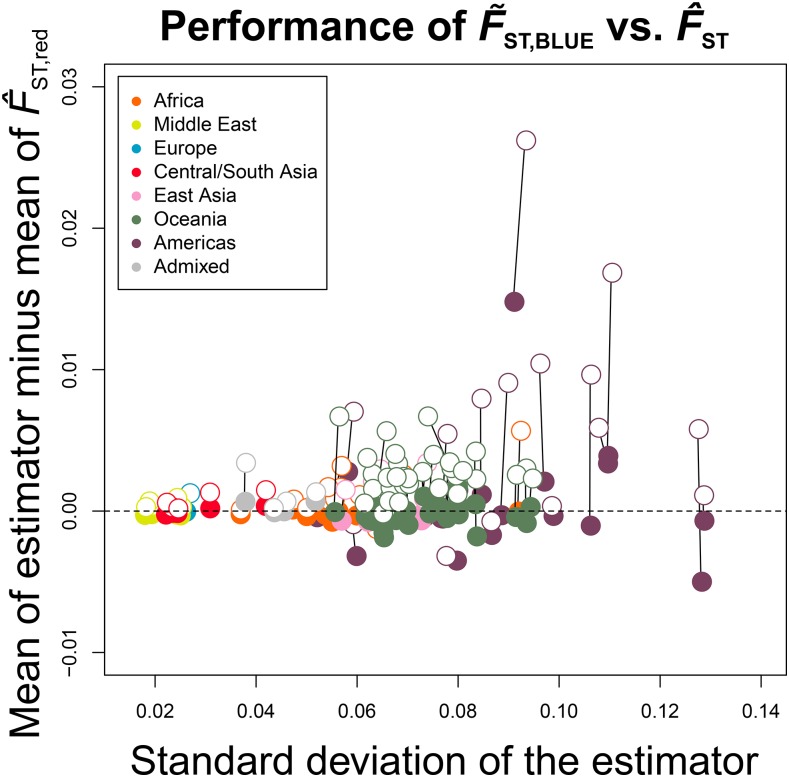
Application of the estimators ${\tilde{H}}_{\text{BLUE}}$ and ${\hat{H}}_{\text{full}}$ to the estimation of $F_{\text{ST}}$ as ${\hat{F}}_{\text{ST}}$ and ${\tilde{F}}_{\text{ST},\text{BLUE}},$ respectively, from empirical data. Similarly to Figure 4, the difference between the mean of the estimator of $F_{\text{ST}}$ (either derived from ${\tilde{H}}_{\text{BLUE}}$ or ${\hat{H}}_{\text{full}}$) and an unbiased estimator (derived from ${\hat{H}}_{\text{red}}$), is displayed on the vertical axis, while the SD of the estimator is displayed on the horizontal axis. The empty circles represent the Reynolds *et al.* (1983) estimator (identical to the ${\hat{H}}_{\text{full}}$-derived estimation), while the filled circles represent the estimation derived from ${\tilde{H}}_{\text{BLUE}}.$ Here, the $F_{\text{ST}}$ values for the French sample with each of the 92 other samples containing related individuals in the dataset MS5795 are plotted, colored by the region of the changing sample.

We additionally find that ${\hat{F}}_{\text{ST}}$ has an upward bias compared with ${\hat{F}}_{\text{ST},\text{red}}$ (calculated with ${\hat{H}}_{\text{red}}$), as well as a larger SD in general than ${\tilde{F}}_{\text{ST},\text{BLUE}}$ (Figure 6). Furthermore, all values of ${\tilde{F}}_{\text{ST},\text{BLUE}}$ are smaller than the paired value of ${\hat{F}}_{\text{ST}}$ calculated for the same population. The difference in the mean of ${\hat{F}}_{\text{ST}}$ and of ${\tilde{F}}_{\text{ST},\text{BLUE}}$ across all loci with the mean of ${\hat{F}}_{\text{ST},\text{red}},$ an estimator which serves as a proxy for the true value of $F_{\text{ST}},$ is displayed on the vertical axis, while the horizontal axis measures the SD of ${\hat{F}}_{\text{ST}}$ and of ${\tilde{F}}_{\text{ST},\text{BLUE}}$ (Figure 6). Supporting our observations indicating the improved accuracy of ${\tilde{F}}_{\text{ST},\text{BLUE}}$ over ${\hat{F}}_{\text{ST}},$ Wilcoxon signed-rank tests (Table 2) between ${\hat{F}}_{\text{ST},\text{red}}$ and either ${\hat{F}}_{\text{ST}}$ or ${\tilde{F}}_{\text{ST},\text{BLUE}}$ indicate that the inclusion of relatives significantly affects the estimate of population differentiation at the α<0.01 significance level. Meanwhile, ${\hat{F}}_{\text{ST},\text{red}}$ and ${\tilde{F}}_{\text{ST},\text{BLUE}}$ are not significantly different in their estimates. These results suggest that the improved properties of ${\tilde{H}}_{\text{BLUE}}$ transfer to the summaries that include it in their calculations.

**Table 2:** Wilcoxon signed-rank test for weighted mean across all loci of ${\hat{F}}_{\text{ST},\text{red}}$ with ${\hat{F}}_{\text{ST}}$ and ${\tilde{F}}_{\text{ST},\text{BLUE}}$ for the French population with the 92 other populations whose samples contained related individuals

Comparison	*P*-Value for Wilcoxon Signed-Rank Test
${\hat{F}}_{\text{ST},\text{red}}$ with ${\hat{F}}_{\text{ST}}$	$5.25\times 10^{-15}$
${\hat{F}}_{\text{ST},\text{red}}$ with ${\tilde{F}}_{\text{ST},\text{BLUE}}$	0.967

## Discussion

We have introduced ${\tilde{H}}_{\text{BLUE}},$ an extension to the estimator ($\tilde{H}$) of expected heterozygosity developed by DeGiorgio *et al.* (2010) that yields a smaller mean squared error in samples containing related individuals, while maintaining unbiasedness. Conveniently, the derivations of ${\tilde{H}}_{\text{BLUE}},$ and its variance, are parallel in form to those of $\tilde{H},$ and we were therefore able to analytically evaluate the performance of the new estimator simultaneously with that of its predecessor. Our updated estimator, ${\tilde{H}}_{\text{BLUE}},$ is based on results from McPeek *et al.* (2004), who characterized the BLUE (${\tilde{p}}_{i}$) of allele frequency. The BLUE improves the precision of allele frequency estimation in complex pedigrees, for which the sample proportion (${\hat{p}}_{i},$ the estimator of allele frequency used in $\hat{H}$ and $\tilde{H}$) is unbiased, but increases in variance with inclusion of related and inbred individuals. Because the properties of the estimator of allele frequency transfer to the estimator of expected heterozygosity, ${\tilde{H}}_{\text{BLUE}}$ is likely to outperform $\tilde{H}$ in situations where ${\tilde{p}}_{i}$ has a smaller variance than ${\hat{p}}_{i}.$ This trend is true for genome-wide data as well (Figure 4 and Table 1).

Overall, ${\tilde{H}}_{\text{BLUE}}$ yields identical results to $\tilde{H}$ in samples containing only one relative pair type, but the two diverge in performance as sample complexity increases (see heat maps in Figure 3, Figure S4, Figure S5, Figure S6, Figure S7, and Figure S8). While both estimators are unbiased, $\tilde{H}$ experiences a larger increase in variance for each additional relative pair type introduced into a sample after the first. This holds true for all sample types regardless of ploidy and inbreeding, suggesting that ${\tilde{H}}_{\text{BLUE}}$ will outperform $\tilde{H}$ in practice, where datasets are often complex. Furthermore, the results of our empirical analysis provide an equally important complement to this observation. Of the 93 populations from the MS5795 dataset we considered that contained relative pairs in their samples, each contained sampled individuals that were not related to any other in the sample. Thus, these samples were more complex than those in which each individual was part of a relative pair of the same type. For most of these cases, except for some American populations (discussed below), ${\tilde{H}}_{\text{BLUE}}$ outperformed $\tilde{H}.$ This is corroborated by the Wilcoxon signed-rank test (Table 1). We expect therefore that any scenario in which there is heterogeneity in relative pair type among sampled individuals, as is observed in many human population-genetic datasets (Pemberton *et al.* 2010, 2013), should favor the application of ${\tilde{H}}_{\text{BLUE}}$ over other estimators.

In addition, random sampling of small isolated populations yields an increased chance that related individuals will be included with large enough sample sizes. Further, inbreeding may confound estimates of diversity, and mislead ${\hat{H}}_{\text{full}}$ to underreport true population expected heterozygosity. Populations of interest that may display these attributes include geographically isolated human settlements in remote alpine (Coia *et al.* 2012; Capocasa *et al.* 2013), South American rainforest (Wang *et al.* 2007), and Siberian taiga and steppe habitats (Dulik *et al.* 2012), and groups such as the Old Order Amish (Van Hout *et al.* 2010), Hutterites (Abney *et al.* 2002; Chong *et al.* 2011), and Mennonites (Payne *et al.* 2011). Further, though our analysis did not directly consider polyploid organisms, the applicability of ${\tilde{H}}_{\text{BLUE}}$ to samples containing individuals of any, and varying, ploidy highlights its usefulness for such data. Prominently, analysis on polyploid organisms such as plants including tetraploid *Arabidopsis thaliana* (Hollister *et al.* 2012), and hexaploid bread wheat (Nielsen *et al.* 2014), both of which self-fertilize, and may therefore be inbred, as well as commercially and ecologically significant Hymenopteran insects, including honeybees (Solignac *et al.* 2003; Harpur *et al.* 2014), bumblebees (Lye *et al.* 2011), and ants (Butler *et al.* 2014), whose males are haploid at all loci, while females are diploid, is likely to benefit from the improved accuracy and precision of ${\tilde{H}}_{\text{BLUE}}.$

We additionally believe that continued investigations into the diversity at single sites in organisms as diverse as dogs (Sutter *et al.* 2007), gray wolves (Zhang *et al.* 2014), humans living at high altitude (Simonson *et al.* 2010; Huerta-Sánchez *et al.* 2013), and rice (Huang *et al.* 2012), in addition to host-microbiome studies (Blekhman *et al.* 2015), will benefit from the advances provided by ${\tilde{H}}_{\text{BLUE}}.$ These studies, as well as many others, have performed scans for positive selection using genomic outliers of population differentiation-based statistics (*e.g.*, $F_{\text{ST}},$ locus-specific branch length, and the population branch statistic), where the calculation is performed per-site, rather than averaged across a large number of sites. Such studies would benefit from estimators of genetic diversity, such as ${\tilde{H}}_{\text{BLUE}}$ and ${\tilde{F}}_{\text{ST},\text{BLUE}},$ with improved variance.

It is pertinent at this point to revisit a pair of potential limitations in our method and examine their implications. First, in Figure 4 (rightmost panel), the mean of $\tilde{H}$ is either closer to that of ${\hat{H}}_{\text{red}}$ than to ${\tilde{H}}_{\text{BLUE}},$ has smaller SD than ${\tilde{H}}_{\text{BLUE}},$ or both for certain samples (predominantly from the Americas). These observations indicate that the accuracy and precision of ${\tilde{H}}_{\text{BLUE}}$ may be impacted by the accuracy of the kinship information incorporated into the calculation. The pedigrees of smaller, more remotely located, populations may be more complex compared to those of larger groups. Further, with a greater proportion of relative pairs in each sample, the effect of relative pair type misidentification may be larger. For RELPAIR (Epstein *et al.* 2000), which was the software chosen to identify relative pairs in MS5795 samples, second-degree pairs cannot be identified as confidently as first-degree pairs (Pemberton *et al.* 2010). Even so, although $\tilde{H}$ may exhibit a somewhat greater robustness to relative pair misclassification, it is still generally outperformed by ${\tilde{H}}_{\text{BLUE}}.$

The second point we address is the smaller MSE of ${\hat{H}}_{\text{full}}$ at less diverse loci in the dataset, especially for samples with fewer relative pairs. While the variance of ${\hat{H}}_{\text{full}}$ is always smaller than that of the other estimators, its bias increases with increasing locus allelic diversity. It is for this reason that the unbiasedness of ${\tilde{H}}_{\text{BLUE}}$ is its most desirable property. In practice, the mean of expected heterozygosity is often taken across loci. Based on such an approach, ${\tilde{H}}_{\text{BLUE}}$ (and $\tilde{H}$ as well) will return the mean expected heterozygosity, and the variance of this estimation (as with all estimators taking the mean across loci) approaches zero as more loci are sampled. An interesting property of all estimators is that their variance (and therefore MSE) is larger for loci whose value for *B* is closer to 1, where B=D/R (B∈[0,1]; see *Results* and Figure S3). Because this effect is greatest for loci with lower true values of *H*, we expect ${\hat{H}}_{\text{full}}$ to have the smallest MSE of all estimators at less diverse loci that are close to their maximum expected heterozygosity, and for which the sample mean kinship coefficient is insufficiently large to appreciably bias the estimator (Equation 12). It is thus important to note that no estimator is uniformly superior to the others. Accordingly, the unique limitation of ${\tilde{H}}_{\text{BLUE}}$ is that the sample kinship matrix must be invertible for the calculation to proceed.

${\tilde{H}}_{\text{BLUE}}$ additionally confers its improved MSE over ${\hat{H}}_{\text{full}}$ downstream to calculations that incorporate estimates of expected heterozygosity. To illustrate this point, we computed $F_{\text{ST}}$ as a function of three estimators: ${\hat{H}}_{\text{full}},$ $\tilde{H},$ and ${\tilde{H}}_{\text{BLUE}}.$ For simulated data, we found that ${\tilde{F}}_{\text{ST},\text{BLUE}},$ yielded an estimate with smaller MSE for the three tested loci than did ${\hat{F}}_{\text{ST}}$ (Figure 5) or ${\tilde{F}}_{\text{ST}},$ and a much smaller mean distance from the true $F_{\text{ST}}$ value than ${\hat{F}}_{\text{ST}}.$ For empirical data (Figure 6), we observed a consistent upward bias for ${\hat{F}}_{\text{ST}}$ compared to ${\hat{F}}_{\text{ST},\text{red}}$ in samples containing relatives that followed much the same pattern as the downward bias of ${\hat{H}}_{\text{full}}$ for such samples. This trend is clear when we consider the formula for $F_{\text{ST}},$ which can be written as $1-\left(H_{1}+H_{2}\right)/\left(2H_{12}\right).$ Taking ${\hat{H}}_{1,\text{full}}$ and ${\hat{H}}_{2,\text{full}}$ as $H_{1}$ and $H_{2},$ this expression yields a larger value than if ${\hat{H}}_{1,\text{red}}$ and ${\hat{H}}_{2,\text{red}}$ were used, because the ratio $\left(H_{1}+H_{2}\right)/\left(2H_{12}\right)$ is smaller for downwardly biased estimators. Interestingly, the SD of ${\tilde{F}}_{\text{ST},\text{BLUE}}$ is, in most cases, smaller than that of ${\hat{F}}_{\text{ST}}$ for the dataset, while the SD of ${\tilde{H}}_{\text{BLUE}}$ was frequently (though not consistently) larger than that of ${\hat{H}}_{\text{full}}$ (Figure 4, center panel).

It is thus noteworthy to consider that the performance of ${\tilde{H}}_{\text{BLUE}}$ and ${\hat{H}}_{\text{full}}$ may diverge further in their applications, where any improvement in MSE for ${\tilde{H}}_{\text{BLUE}}$ may be magnified downstream. This is highlighted by the increased concordance between ${\tilde{F}}_{\text{ST},\text{BLUE}}$ and ${\hat{F}}_{\text{ST},\text{red}}$ compared to ${\tilde{H}}_{\text{BLUE}}$ and ${\hat{H}}_{\text{red}}$ (*cf*. *P*-values between Table 1 and Table 2). With this in mind, applications of ${\tilde{F}}_{\text{ST},\text{BLUE}}$ can also be considered. Two such examples are the locus-specific branch length (LSBL; Shriver *et al.* 2004) and the similar population branch statistic (PBS; Yi *et al.* 2010). These statistics incorporate $F_{\text{ST}}$ values between three populations as measures of branch length to detect positive selection at a locus. Loci for which the unrooted three-taxon tree indicates a significantly longer branch length in a particular lineage may represent regions possibly under selection. To allow for the easy application of ${\tilde{H}}_{\text{BLUE}},$ we have written an R script, *BestHet*, that computes ${\tilde{H}}_{\text{BLUE}},$ ${\tilde{F}}_{\text{ST},\text{BLUE}},$ and $LSBL_{\text{BLUE}},$ given matrices of genotype and kinship data for a sample (download available at http://www.personal.psu.edu/mxd60/best_het.html).

## Supplementary Material

Supplemental material is available online at www.g3journal.org/lookup/suppl/doi:10.1534/g3.116.037168/-/DC1.

## Appendix

### Derivations of unbiased estimators of expected heterozygosity

In this section, we derive the general unbiased estimator of expected heterozygosity $\overset{˘}{H}$ for any unbiased linear estimator of population allele frequencies, defined in Proposition 1, and show how the formulas for $\tilde{H}$ (DeGiorgio *et al.* 2010), and ${\tilde{H}}_{\text{BLUE}}$ (Corollaries 2 and 3), emerge from specific cases of $\overset{˘}{H}.$

#### Proof of Proposition 1:

We need to show that $E\left[\overset{˘}{H}\right]=H.$ Note that

$$\begin{array}{c} {\overset{˘}{p}}_{i}^{2}=\sum_{j=1}^{n}\sum_{k=1}^{n}w_{j}w_{k}X_{j}^{\left(i\right)}X_{k}^{\left(i\right)}\\ =\sum_{j=1}^{n}\sum_{k=1}^{n}\frac{w_{j}w_{k}}{m_{j}m_{k}}\sum_{\ell =1}^{m_{j}}\sum_{t=1}^{m_{k}}A_{j\ell }^{\left(i\right)}A_{kt}^{\left(i\right)}. \end{array}$$

Taking the expectation, we obtain

$$\begin{array}{c} E\left[{\overset{˘}{p}}_{i}^{2}\right]=\sum_{j=1}^{n}\sum_{k=1}^{n}\frac{w_{j}w_{k}}{m_{j}m_{k}}\sum_{\ell =1}^{m_{j}}\sum_{t=1}^{m_{k}}E\left[A_{j\ell }^{\left(i\right)}A_{kt}^{\left(i\right)}\right]\\ =\sum_{j=1}^{n}\sum_{k=1}^{n}\frac{w_{j}w_{k}}{m_{j}m_{k}}\sum_{\ell =1}^{m_{j}}\sum_{t=1}^{m_{k}}\mathbb{P}\left[A_{j\ell }^{\left(i\right)}=1,A_{kt}^{\left(i\right)}=1\right]\\ =\sum_{j=1}^{n}\sum_{k=1}^{n}\frac{w_{j}w_{k}}{m_{j}m_{k}}\sum_{\ell =1}^{m_{j}}\sum_{t=1}^{m_{k}}\left[\left(1-\Phi_{jk}\right)p_{i}^{2}+\Phi_{jk}p_{i}\right]\\ =p_{i}^{2}+\rho_{2}p_{i}\left(1-p_{i}\right). \end{array}$$ (A1)

Therefore

$$\begin{array}{c} E\left[\overset{˘}{H}\right]=\frac{1}{1-\rho_{2}}\left(1-\sum_{i=1}^{I}E\left[{\overset{˘}{p}}_{i}^{2}\right]\right)\\ =\frac{1}{1-\rho_{2}}\left(1-\sum_{i=1}^{I}\left[p_{i}^{2}+\rho_{2}p_{i}\left(1-p_{i}\right)\right]\right)\\ =1-\sum_{i=1}^{I}p_{i}^{2}\\ =H. \end{array}$$ □

#### Proof of Corollary 2:

We show that defining the weight of each individual in the calculation of $\rho_{2}$ in terms of an individual’s relative allele copy contribution yields $\tilde{H}$ from $\overset{˘}{H}.$ Letting $w_{k}=m_{k}/\sum_{x=1}^{n}m_{x},$ we have that

$$\begin{array}{c} {\overset{˘}{p}}_{i}=\sum_{k=1}^{n}\frac{m_{k}}{\sum_{j=1}^{n}m_{j}}X_{k}^{\left(i\right)}\\ ={\hat{p}}_{i} \end{array}$$

and

$$\begin{array}{c} \rho_{2}=\sum_{j=1}^{n}\sum_{k=1}^{n}\frac{m_{j}}{\sum_{x=1}^{n}m_{x}}\frac{m_{k}}{\sum_{y=1}^{n}m_{y}}\Phi_{jk}\\ ={\bar{\Phi }}_{2}. \end{array}$$

Plugging in yields

$$\overset{˘}{H}=\frac{1}{1-{\bar{\Phi }}_{2}}\left(1-\sum_{i=1}^{I}{\hat{p}}_{i}^{2}\right)=\tilde{H}.$$ □

#### Proof of Corollary 3:

We show that defining the weight each individual according to their relative contribution to the inverted kinship matrix of the sample yields ${\tilde{H}}_{\text{BLUE}}$ from $\overset{˘}{H}.$ Letting $w_{k}={\sum^{\text{​}}}_{j=1}^{n}{\left(K^{-1}\right)}_{jk}/1^{T}K^{-1}1,$ we have that

$$\begin{array}{c} {\overset{˘}{p}}_{i}=\sum_{k=1}^{n}\frac{\sum_{j=1}^{n}{\left(K^{-1}\right)}_{jk}}{1^{T}K^{-1}1}X_{k}^{\left(i\right)}\\ ={\tilde{p}}_{i} \end{array}$$

and

$$\begin{array}{c} \rho_{2}=\sum_{j=1}^{n}\sum_{k=1}^{n}\frac{\sum_{x=1}^{n}{\left(K^{-1}\right)}_{xj}}{1^{T}K^{-1}1}\frac{\sum_{y=1}^{n}{\left(K^{-1}\right)}_{yk}}{1^{T}K^{-1}1}\Phi_{jk}\\ =\kappa_{2}. \end{array}$$

Plugging in yields

$$\begin{array}{c} \overset{˘}{H}=\frac{1}{1-\kappa_{2}}\left(1-\sum_{i=1}^{I}{\tilde{p}}_{i}^{2}\right)\\ ={\tilde{H}}_{\text{BLUE}}. \end{array}$$ □

### Derivations of variances of expected heterozygosity estimators

In this section, we summarize the procedure by which DeGiorgio *et al.* (2010) derived the equation for the variance of $\tilde{H},$ illustrating the variance of the general case, $\overset{˘}{H}.$ For the full derivation, see Appendix B of DeGiorgio *et al.* (2010). We then provide the specific formulation for the variance of $\tilde{H}$ (Corollary 7) and ${\tilde{H}}_{\text{BLUE}}$ (Corollary 8).

#### Abbreviated proof of Proposition 4:

The variance of $\overset{˘}{H}$ (Equation 9) is defined as

$$\text{Var}\left[\overset{˘}{H}\right]=\frac{1}{{\left(1-\rho_{2}\right)}^{2}}\text{Var}\left[1-\sum_{i=1}^{I}{\overset{˘}{p}}_{i}^{2}\right].$$

By definition of variance, we get

$$\text{Var}\left[1-\sum_{i=1}^{I}{\overset{˘}{p}}_{i}^{2}\right]=\sum_{i=1}^{I}\text{Var}\left[{\overset{˘}{p}}_{i}^{2}\right]+2\sum_{i=1}^{I-1}\sum_{i^{\prime }=i+1}^{I}\text{Cov}\left[{\overset{˘}{p}}_{i}^{2},{\overset{˘}{p}}_{i^{\prime }}^{2}\right],$$

with

$$\text{Var}\left[{\overset{˘}{p}}_{i}^{2}\right]=E\left[{\overset{˘}{p}}_{i}^{4}\right]-{\left(E\left[{\overset{˘}{p}}_{i}^{2}\right]\right)}^{2}$$

and

$$\text{Cov}\left[{\overset{˘}{p}}_{i}^{2},{\overset{˘}{p}}_{i^{\prime }}^{2}\right]=E\left[{\overset{˘}{p}}_{i}^{2}{\overset{˘}{p}}_{i^{\prime }}^{2}\right]-E\left[{\overset{˘}{p}}_{i}^{2}\right]E\left[{\overset{˘}{p}}_{i^{\prime }}^{2}\right].$$

Recalling that ${\overset{˘}{p}}_{i}=\sum_{j=1}^{n}\sum_{\ell =1}^{m_{j}}\frac{w_{j}}{m_{j}}A_{j\ell }^{\left(i\right)}$ for the ℓth allele copy of individual *j*, whose ploidy is $m_{j},$ we have that

$$E\left[{\overset{˘}{p}}_{i}^{4}\right]=\sum_{j=1}^{n}\sum_{k=1}^{n}\sum_{j^{\prime }=1}^{n}\sum_{k^{\prime }=1}^{n}\sum_{\ell =1}^{m_{j}}\sum_{t=1}^{m_{k}}\sum_{\ell^{\prime }=1}^{m_{j^{\prime }}}\sum_{t^{\prime }=1}^{m_{k^{\prime }}}\frac{w_{j}w_{k}w_{j\prime }w_{k\prime }}{m_{j}m_{k}m_{j\prime }m_{k\prime }}E\left[A_{j\ell }^{\left(i\right)}A_{kt}^{\left(i\right)}A_{j\prime \ell \prime }^{\left(i\right)}A_{k\prime t\prime }^{\left(i\right)}\right],$$

and

$$E\left[{\overset{˘}{p}}_{i}^{2}{\overset{˘}{p}}_{i\prime }^{2}\right]=\sum_{j=1}^{n}\sum_{k=1}^{n}\sum_{j^{\prime }=1}^{n}\sum_{k^{\prime }=1}^{n}\sum_{\ell =1}^{m_{j}}\sum_{t=1}^{m_{k}}\sum_{\ell^{\prime }=1}^{m_{j^{\prime }}}\sum_{t^{\prime }=1}^{m_{k^{\prime }}}\frac{w_{j}w_{k}w_{j^{\prime }}w_{k^{\prime }}}{m_{j}m_{k}m_{j^{\prime }}m_{k^{\prime }}}E\left[A_{j\ell }^{\left(i\right)}A_{kt}^{\left(i\right)}A_{j^{\prime }\ell^{\prime }}^{\left(i^{\prime }\right)}A_{k^{\prime }t^{\prime }}^{\left(i^{\prime }\right)}\right]$$

for the case $i\neq i^{\prime }.$ We have previously shown in Equation A1 that $E\left[{\overset{˘}{p}}_{i}^{2}\right]=p_{i}^{2}+\rho_{2}p_{i}\left(1-p_{i}\right),$ and the value of $E\left[{\overset{˘}{p}}_{i^{\prime }}^{2}\right]$ similarly follows. Thus, we need to calculate $E\left[A_{j\ell }^{\left(i\right)}A_{kt}^{\left(i\right)}A_{j^{\prime }\ell^{\prime }}^{\left(i\right)}A_{k^{\prime }t^{\prime }}^{\left(i\right)}\right]$ and $E\left[A_{j\ell }^{\left(i\right)}A_{kt}^{\left(i\right)}A_{j^{\prime }\ell^{\prime }}^{\left(i^{\prime }\right)}A_{k^{\prime }t^{\prime }}^{\left(i^{\prime }\right)}\right]$ for the $i\neq i^{\prime }$ case. These are

$$\begin{array}{c} E\left[A_{j\ell }^{\left(i\right)}A_{kt}^{\left(i\right)}A_{j^{\prime }\ell^{\prime }}^{\left(i\right)}A_{k^{\prime }t^{\prime }}^{\left(i\right)}\right]=\Phi_{jkj^{\prime }k^{\prime }}p_{i}+\left[\Phi_{jkj^{\prime }}+\Phi_{jkk^{\prime }}+\Phi_{jj^{\prime }k^{\prime }}+\Phi_{kj^{\prime }k^{\prime }}+\Phi_{jk,j^{\prime }k^{\prime }}+\Phi_{jj^{\prime },kk^{\prime }}+\Phi_{jk^{\prime },kj^{\prime }}-7\Phi_{jkj^{\prime }k^{\prime }}\right]p_{i}^{2}\\ +[12\Phi_{jkj^{\prime }k^{\prime }}+\Phi_{jk}+\Phi_{jj^{\prime }}+\Phi_{jk^{\prime }}+\Phi_{kj^{\prime }}+\Phi_{kk^{\prime }}+\Phi_{j^{\prime }k^{\prime }}-3\left(\Phi_{jkj^{\prime }}+\Phi_{jkk^{\prime }}+\Phi_{jj^{\prime }k^{\prime }}+\Phi_{kj^{\prime }k^{\prime }}\right)\\ -2\left(\Phi_{jk,j^{\prime }k^{\prime }}+\Phi_{jj,kk^{\prime }}+\Phi_{jk,kj^{\prime }}\right)]p_{i}^{3}+[1+\Phi_{jk,j^{\prime }k^{\prime }}+\Phi_{jj^{\prime },kk^{\prime }}+\Phi_{jk^{\prime },kj^{\prime }}\\ +2\left(\Phi_{jkj^{\prime }}+\Phi_{jkk^{\prime }}+\Phi_{jj^{\prime }k^{\prime }}+\Phi_{kj^{\prime }k^{\prime }}\right)-6\Phi_{jkj^{\prime }k^{\prime }}-\left(\Phi_{jk}+\Phi_{jj^{\prime }}+\Phi_{jk^{\prime }}+\Phi_{kj^{\prime }}+\Phi_{kk^{\prime }}+\Phi_{j^{\prime }k^{\prime }}\right)]p_{i}^{4} \end{array}$$ (A2)

and

$$\begin{array}{c} E\left[A_{j\ell }^{\left(i\right)}A_{kt}^{\left(i\right)}A_{j^{\prime }\ell^{\prime }}^{\left(i^{\prime }\right)}A_{k^{\prime }t^{\prime }}^{\left(i^{\prime }\right)}\right]=\left[\Phi_{jk,j^{\prime }k^{\prime }}-\Phi_{jkj^{\prime }k^{\prime }}\right]p_{i}p_{i^{\prime }}+\left[2\Phi_{jkj^{\prime }k^{\prime }}+\Phi_{jk}-\left(\Phi_{jkj^{\prime }}+\Phi_{jkk^{\prime }}\right)-\Phi_{jk,j^{\prime }k^{\prime }}\right]p_{i}p_{i^{\prime }}^{2}\\ +\left[2\Phi_{jkj^{\prime }k^{\prime }}+\Phi_{j^{\prime }k^{\prime }}-\left(\Phi_{jj^{\prime }k^{\prime }}+\Phi_{kj^{\prime }k^{\prime }}\right)-\Phi_{jk,j^{\prime }k^{\prime }}\right]p_{i}^{2}p_{i^{\prime }}+[1+\Phi_{jk,j^{\prime }k^{\prime }}+\Phi_{jj^{\prime },kk^{\prime }}+\Phi_{jk^{\prime },kj^{\prime }}\\ +2\left(\Phi_{jkj^{\prime }}+\Phi_{jkk^{\prime }}+\Phi_{jj^{\prime }k^{\prime }}+\Phi_{kj^{\prime }k^{\prime }}\right)-6\Phi_{jkj^{\prime }k^{\prime }}-\left(\Phi_{jk}+\Phi_{jj^{\prime }}+\Phi_{jk^{\prime }}+\Phi_{kj^{\prime }}+\Phi_{kk^{\prime }}+\Phi_{j^{\prime }k^{\prime }}\right)]p_{i}^{2}p_{i^{\prime }}^{2}. \end{array}$$ (A3)

Substituting Equation A2 into $E\left[{\overset{˘}{p}}_{i}^{4}\right]$ and solving for $\text{Var}\left[{\overset{˘}{p}}_{i}^{2}\right],$ we obtain

$$\text{Var}\left[{\overset{˘}{p}}_{i}^{2}\right]=\rho_{4}p_{i}+\left(4\rho_{3}+3\rho_{2,2}-7\rho_{4}-\rho_{2}^{2}\right)p_{i}^{2}+\left(12\rho_{4}+4\rho_{2}+2\rho_{2}^{2}-12\rho_{3}-6\rho_{2,2}\right)p_{i}^{3}+\left(3\rho_{2,2}+8\rho_{3}-6\rho_{4}-4\rho_{2}-\rho_{2}^{2}\right)p_{i}^{4},$$

and, substituting Equation A3 into $E\left[{\overset{˘}{p}}_{i}^{2}{\overset{˘}{p}}_{i\prime }^{2}\right],$ and solving for $\text{Cov}\left[{\overset{˘}{p}}_{i}^{2},{\overset{˘}{p}}_{i^{\prime }}^{2}\right],$ we obtain

$$\text{Cov}\left[{\overset{˘}{p}}_{i}^{2},{\overset{˘}{p}}_{i^{\prime }}^{2}\right]=\left(\rho_{2,2}-\rho_{4}-\rho_{2}^{2}\right)p_{i}p_{i^{\prime }}+\left(2\rho_{4}+\rho_{2}^{2}-2\rho_{3}-\rho_{2,2}\right)p_{i}p_{i^{\prime }}^{2}+\left(2\rho_{4}+\rho_{2}^{2}-2\rho_{3}-\rho_{2,2}\right)p_{i}^{2}p_{i^{\prime }}+\left(3\rho_{2,2}+8\rho_{3}-6\rho_{4}-4\rho_{2}-\rho_{2}^{2}\right)p_{i}^{2}p_{i^{\prime }}^{2}.$$

Thus, substituting the values for variance and covariance into the definition of variance, we have

$$\begin{array}{l} \text{Var}\left[\overset{˘}{H}\right]=\frac{1}{{\left(1-\rho_{2}\right)}^{2}}[\rho_{2,2}-\rho_{2}^{2}+2\left(\rho_{2}^{2}-\rho_{4}\right)\sum_{i=1}^{I}p_{i}^{2}+4\left(2\rho_{4}+\rho_{2}-2\rho_{3}-\rho_{2,2}\right)\sum_{i=1}^{I}p_{i}^{3}\\ +\left(3\rho_{2,2}+8\rho_{3}-6\rho_{4}-4\rho_{2}-\rho_{2}^{2}\right){\left(\sum_{i=1}^{I}p_{i}^{2}\right)}^{2}]. \end{array}$$ □

#### Corollary 7:

Consider a locus with *I* distinct alleles, and parametric allele frequencies $p_{i}\in \left[0,1\right],$ i=1,2,…,I, and $\sum_{i=1}^{I}p_{i}=1.$ For a sample of size *n* individuals of any ploidy, inbreeding status, and relatedness,

$$\text{Var}\left[\tilde{H}\right]=\frac{1}{{\left(1-{\bar{\Phi }}_{2}\right)}^{2}}\text{Var}\left[1-\sum_{i=1}^{I}{\hat{p}}_{i}^{2}\right]$$ (A4)

and

$$\begin{array}{c} \text{Var}\left[1-\sum_{i=1}^{I}{\hat{p}}_{i}^{2}\right]={\bar{\Phi }}_{2,2}-{\bar{\Phi }}_{2}^{2}+2\left({\bar{\Phi }}_{2}^{2}-{\bar{\Phi }}_{4}\right)\sum_{i=1}^{I}p_{i}^{2}+4\left(2{\bar{\Phi }}_{4}+{\bar{\Phi }}_{2}-2{\bar{\Phi }}_{3}-{\bar{\Phi }}_{2,2}\right)\sum_{i=1}^{I}p_{i}^{3}\\ +\left(3{\bar{\Phi }}_{2,2}+8{\bar{\Phi }}_{3}-6{\bar{\Phi }}_{4}-4{\bar{\Phi }}_{2}-{\bar{\Phi }}_{2}^{2}\right){\left(\sum_{i=1}^{I}p_{i}^{2}\right)}^{2}, \end{array}$$ (A5)

where ${\bar{\Phi }}_{2},$ ${\bar{\Phi }}_{3},$ ${\bar{\Phi }}_{4},$ and ${\bar{\Phi }}_{2,2}$ are mean kinship coefficients, weighted by the contribution of individuals to the number of allele copies in the sample, with subscripts corresponding to the number of individuals considered for the calculation. Additionally,

$$\text{Var}\left[\tilde{H}\right]\approx 4{\bar{\Phi }}_{2}\left[\sum_{i=1}^{I}p_{i}^{3}-{\left(\sum_{i=1}^{I}p_{i}^{2}\right)}^{2}\right].$$ (A6)

The proof of Corollary 7 follows from the proof of Proposition 4, where ${\hat{p}}_{i}$ is substituted for ${\overset{˘}{p}}_{i},$ and ${\bar{\Phi }}_{2},$ ${\bar{\Phi }}_{3},$ ${\bar{\Phi }}_{4},$ and ${\bar{\Phi }}_{2,2}$ are substituted for $\rho_{2},$ $\rho_{3},$ $\rho_{4},$ and $\rho_{2,2},$ respectively.

#### Corollary 8:

Consider a locus with *I* distinct alleles and parametric allele frequencies $p_{i}\in \left[0,1\right],$ i=1,2,…,I, and $\sum_{i=1}^{I}p_{i}=1.$ For a sample of size *n* individuals of any ploidy, inbreeding status, and relatedness,

$$\text{Var}\left[{\tilde{H}}_{\text{BLUE}}\right]=\frac{1}{{\left(1-\kappa_{2}\right)}^{2}}\text{Var}\left[1-\sum_{i=1}^{I}{\tilde{p}}_{i}^{2}\right]$$ (A7)

and

$$\begin{array}{c} \text{Var}\left[1-\sum_{i=1}^{I}{\tilde{p}}_{i}^{2}\right]=\kappa_{2,2}-\kappa_{2}^{2}+2\left(\kappa_{2}^{2}-\kappa_{4}\right)\sum_{i=1}^{I}p_{i}^{2}+4\left(2\kappa_{4}+\kappa_{2}-2\kappa_{3}-\kappa_{2,2}\right)\sum_{i=1}^{I}p_{i}^{3}\\ +\left(3\kappa_{2,2}+8\kappa_{3}-6\kappa_{4}-4\kappa_{2}-\kappa_{2}^{2}\right){\left(\sum_{i=1}^{I}p_{i}^{2}\right)}^{2}, \end{array}$$ (A8)

where $\kappa_{2},$ $\kappa_{3},$ $\kappa_{4},$ and $\kappa_{2,2}$ are mean kinship coefficients, weighted by the contribution of individuals to the inverted kinship matrix, with subscripts corresponding to the number of individuals considered for the calculation. Additionally,

$$\text{Var}\left[{\tilde{H}}_{\text{BLUE}}\right]\approx 4\kappa_{2}\left[\sum_{i=1}^{I}p_{i}^{3}-{\left(\sum_{i=1}^{I}p_{i}^{2}\right)}^{2}\right].$$ (A9)

The proof of Corollary 8 follows from the proof of Proposition 4, where ${\tilde{p}}_{i}$ is substituted for ${\overset{˘}{p}}_{i},$ and $\kappa_{2},$ $\kappa_{3},$ $\kappa_{4},$ and $\kappa_{2,2}$ are substituted for $\rho_{2},$ $\rho_{3},$ $\rho_{4},$ and $\rho_{2,2},$ respectively.

### Derivations of bias measurements in the application of $\hat{\text{H}}$

For samples containing related and inbred individuals, $\hat{H}$ has a downward bias, which is defined in Equation 12 for the general estimator of population allele frequency ${\overset{˘}{p}}_{i}.$ Here, we present Corollaries 9 and 10 for the specific estimators of population allele frequency ${\hat{p}}_{i}$ and ${\tilde{p}}_{i},$ respectively.

#### Corollary 9:

Consider a locus with *I* distinct alleles and parametric allele frequencies $p_{i}\in \left[0,1\right],$ i=1,2,…,I, and $\sum_{i=1}^{I}p_{i}=1.$ For a sample of size *n* possibly related or inbred individuals, the bias of the estimator of expected heterozygosity $\hat{H}$ changes with the true locus expected heterozygosity such that

$$\text{Bias}\left[\hat{H}\left({\hat{p}}_{i}\right)\right]=\frac{1-n{\bar{\Phi }}_{2}}{n-1}H,$$ (A10)

where

$$\hat{H}\left({\hat{p}}_{i}\right)=\frac{n}{n-1}\left(1-\sum_{i=1}^{I}{\hat{p}}_{i}^{2}\right).$$

As this is the standard application of $\hat{H}$ (Equation 2), Equation A10 describes the bias of $\hat{H}$ in the *Results*. However, $\hat{H}$ is biased with any unbiased linear estimator of allele frequency for samples containing related or inbred individuals. The proof of Corollary 9 follows from the proof of Proposition 5, where ${\bar{\Phi }}_{2}$ is substituted for $\rho_{2}.$

#### Corollary 10:

Consider a locus with *I* distinct alleles and parametric allele frequencies $p_{i}\in \left[0,1\right],$ i=1,2,…,I, and $\sum_{i=1}^{I}p_{i}=1.$ For a sample of size *n* possibly related or inbred individuals, the bias of the estimator of expected heterozygosity $\hat{H}$ changes with the true locus expected heterozygosity such that

$$\text{Bias}\left[\hat{H}\left({\tilde{p}}_{i}\right)\right]=\frac{1-n\kappa_{2}}{n-1}H,$$ (A11)

where

$$\hat{H}\left({\tilde{p}}_{i}\right)=\frac{n}{n-1}\left(1-\sum_{i=1}^{I}{\tilde{p}}_{i}^{2}\right).$$ (A12)

The proof of Corollary 10 follows from the proof of Proposition 5, where $\kappa_{2}$ is substituted for $\rho_{2}.$

### Derivations of components for the variance of ${\text{F}}_{\text{ST}}$ estimators

In this final section of the Appendix, we provide derivations for the components of Equations 16 and 17, which describe the variance of ${\overset{˘}{F}}_{\text{ST}}.$ We derive the variance of ${\overset{˘}{H}}_{12},$ as well as the covariances of ${\overset{˘}{H}}_{12}$ with ${\overset{˘}{H}}_{1}$ (and interchangeably, ${\overset{˘}{H}}_{12}$ with ${\overset{˘}{H}}_{2}$), and of $\left[{\overset{˘}{H}}_{12}-\frac{1}{2}{\overset{˘}{H}}_{1}-\frac{1}{2}{\overset{˘}{H}}_{2}\right]$ with ${\overset{˘}{H}}_{12}.$ Because the complete expression for $\text{Var}\left[{\overset{˘}{F}}_{\text{ST}}\right]$ is unwieldy, we stop at the derivation of the final component.

#### Lemma 11:

Consider a locus with *I* distinct alleles across two independent populations and parametric allele frequencies $p_{i}\in \left[0,1\right],$ i=1,2,…,I, and $\sum_{i=1}^{I}p_{i}=1$ for population 1, and $q_{i}\in \left[0,1\right],$ i=1,2,…,I, and $\sum_{i=1}^{I}q_{i}=1$ for population 2. For two samples of size $n_{1}$ and $n_{2},$ individuals from populations 1 and 2, respectively, each with individuals of any ploidy, inbreeding status, and relatedness,

$$\text{Var}\left[{\overset{˘}{H}}_{12}\right]=\rho_{2}^{\left(1\right)}\left(1-\rho_{2}^{\left(2\right)}\right)\sum_{i=1}^{I}p_{i}q_{i}^{2}+\rho_{2}^{\left(2\right)}\left(1-\rho_{2}^{\left(1\right)}\right)\sum_{i=1}^{I}p_{i}^{2}q_{i}+\rho_{2}^{\left(1\right)}\rho_{2}^{\left(2\right)}\sum_{i=1}^{I}p_{i}q_{i}+\left(\rho_{2}^{\left(1\right)}\rho_{2}^{\left(2\right)}-\rho_{2}^{\left(1\right)}-\rho_{2}^{\left(2\right)}\right){\left(\sum_{i=1}^{I}p_{i}q_{i}\right)}^{2},$$ (A13)

where the superscript of the mean kinship coefficient $\rho_{2}$ corresponds to the population for which it is calculated. The equations for the variance of ${\tilde{H}}_{12}$ and ${\tilde{H}}_{12,\text{BLUE}}$ are obtained by substituting ${\bar{\Phi }}_{2}$ and $\kappa_{2},$ respectively, into Equation A13 as the mean kinship coefficients in place of $\rho_{2}.$

#### Proof:

By definition of variance,

$$\text{Var}\left[{\overset{˘}{H}}_{12}\right]=\sum_{i=1}^{I}\text{Var}\left[{\overset{˘}{p}}_{i}{\overset{˘}{q}}_{i}\right]+2\sum_{i=1}^{I-1}\sum_{i^{\prime }=i+1}^{I}\text{Cov}\left[{\overset{˘}{p}}_{i}{\overset{˘}{q}}_{i},{\overset{˘}{p}}_{i^{\prime }}{\overset{˘}{q}}_{i^{\prime }}\right]$$

where

$$\text{Var}\left[{\overset{˘}{p}}_{i}{\overset{˘}{q}}_{i}\right]=E\left[{\overset{˘}{p}}_{i}^{2}{\overset{˘}{q}}_{i}^{2}\right]-{\left(E\left[{\overset{˘}{p}}_{i}{\overset{˘}{q}}_{i}\right]\right)}^{2}$$

and

$$\text{Cov}\left[{\overset{˘}{p}}_{i}{\overset{˘}{q}}_{i},{\overset{˘}{p}}_{i^{\prime }}{\overset{˘}{q}}_{i^{\prime }}\right]=E\left[{\overset{˘}{p}}_{i}{\overset{˘}{q}}_{i}{\overset{˘}{p}}_{i^{\prime }}{\overset{˘}{q}}_{i^{\prime }}\right]-E\left[{\overset{˘}{p}}_{i}{\overset{˘}{q}}_{i}\right]E\left[{\overset{˘}{p}}_{i^{\prime }}{\overset{˘}{q}}_{i^{\prime }}\right].$$

Because ${\overset{˘}{p}}_{i}$ and ${\overset{˘}{q}}_{i}$ are unbiased estimators of population allele frequency, and populations 1 and 2 are independent,

$$E\left[{\overset{˘}{p}}_{i}{\overset{˘}{q}}_{i}\right]=p_{i}q_{i}$$

Similarly, $E\left[{\overset{˘}{p}}_{i^{\prime }}{\overset{˘}{q}}_{i^{\prime }}\right]=p_{i^{\prime }}q_{i^{\prime }}.$ Next, we have

$$\begin{array}{c} E\left[{\overset{˘}{p}}_{i}^{2}{\overset{˘}{q}}_{i}^{2}\right]=E\left[{\overset{˘}{p}}_{i}^{2}\right]E\left[{\overset{˘}{q}}_{i}^{2}\right]\\ =\left[p_{i}^{2}+\rho_{2}^{\left(1\right)}p_{i}\left(1-p_{i}\right)\right]\left[q_{i}^{2}+\rho_{2}^{\left(2\right)}q_{i}\left(1-q_{i}\right)\right]\\ =p_{i}q_{i}\left[p_{i}+\rho_{2}^{\left(1\right)}\left(1-p_{i}\right)\right]\left[q_{i}+\rho_{2}^{\left(2\right)}\left(1-q_{i}\right)\right], \end{array}$$ (A14)

where $E\left[{\overset{˘}{q}}_{i}^{2}\right]$ takes the same form as $E\left[{\overset{˘}{p}}_{i}^{2}\right]$ (Equation A1), except that the resulting weighted mean kinship coefficient $\rho_{2}$ is for population 2, indicated by the superscript. By substituting Equation A14 into $\text{Var}\left[{\overset{˘}{p}}_{i}{\overset{˘}{q}}_{i}\right],$ we have

$$\begin{array}{c} \text{Var}\left[{\overset{˘}{p}}_{i}{\overset{˘}{q}}_{i}\right]=p_{i}q_{i}\left[p_{i}+\rho_{2}^{\left(1\right)}\left(1-p_{i}\right)\right]\left[q_{i}+\rho_{2}^{\left(2\right)}\left(1-q_{i}\right)\right]-{\left(p_{i}q_{i}\right)}^{2}\\ =p_{i}q_{i}\left\{\left[p_{i}+\rho_{2}^{\left(1\right)}\left(1-p_{i}\right)\right]\left[q_{i}+\rho_{2}^{\left(2\right)}\left(1-q_{i}\right)\right]-p_{i}q_{i}\right\}\\ =p_{i}q_{i}\left[\rho_{2}^{\left(1\right)}\left(1-p_{i}\right)q_{i}+\rho_{2}^{\left(2\right)}p_{i}\left(1-q_{i}\right)+\rho_{2}^{\left(1\right)}\rho_{2}^{\left(2\right)}\left(1-p_{i}\right)\left(1-q_{i}\right)\right]. \end{array}$$ (A15)

We now derive an expression for $\text{Cov}\left[{\overset{˘}{p}}_{i}{\overset{˘}{q}}_{i},{\overset{˘}{p}}_{i^{\prime }}{\overset{˘}{q}}_{i^{\prime }}\right].$ Let $B_{kt}^{\left(i\right)}$ be an indicator random variable in population 2 analogous to the indicator random variable $A_{j\ell }^{\left(i\right)},$ which we have previously defined for population 1.

$$\begin{array}{c} E\left[{\overset{˘}{p}}_{i}{\overset{˘}{q}}_{i}{\overset{˘}{p}}_{i^{\prime }}{\overset{˘}{q}}_{i^{\prime }}\right]=E\left[{\overset{˘}{p}}_{i}{\overset{˘}{p}}_{i^{\prime }}\right]E\left[{\overset{˘}{q}}_{i}{\overset{˘}{q}}_{i^{\prime }}\right]\\ =\left(\sum_{j=1}^{n_{1}}\sum_{j^{\prime }=1}^{n_{1}}\sum_{\ell =1}^{m_{j}}\sum_{\ell^{\prime }=1}^{m_{j^{\prime }}}\frac{w_{j}w_{j^{\prime }}}{m_{j}m_{j^{\prime }}}E\left[A_{j\ell }^{\left(i\right)}A_{j^{\prime }\ell^{\prime }}^{\left(i^{\prime }\right)}\right]\right)\left(\sum_{k=1}^{n_{2}}\sum_{k^{\prime }=1}^{n_{2}}\sum_{t=1}^{m_{k}}\sum_{t^{\prime }=1}^{m_{k^{\prime }}}\frac{w_{k}w_{k^{\prime }}}{m_{k}m_{k^{\prime }}}E\left[B_{kt}^{\left(i\right)}B_{k^{\prime }t^{\prime }}^{\left(i^{\prime }\right)}\right]\right), \end{array}$$

where

$$E\left[A_{j\ell }^{\left(i\right)}A_{j^{\prime }\ell^{\prime }}^{\left(i^{\prime }\right)}\right]=\mathbb{P}\left[A_{j\ell }^{\left(i\right)}=1,A_{j^{\prime }\ell^{\prime }}^{\left(i^{\prime }\right)}=1\right],$$

and

$$E\left[B_{kt}^{\left(i\right)}B_{k^{\prime }t^{\prime }}^{\left(i\prime \right)}\right]=\mathbb{P}\left[B_{kt}^{\left(i\right)}=1,B_{k^{\prime }t^{\prime }}^{\left(i^{\prime }\right)}=1\right].$$

Consider a scenario in which we have two allele copies. Let $s_{1}$ be the identity state with probability $\Delta_{1},$ in which two randomly drawn alleles are not IBD, and $s_{2}$ be the identity state occurring with probability $\Delta_{2}=1-\Delta_{1},$ in which the two alleles are IBD.

$$\begin{array}{c} \mathbb{P}\left[A_{j\ell }^{\left(i\right)}=1,A_{j^{\prime }\ell^{\prime }}^{\left(i^{\prime }\right)}=1\right]=\mathbb{P}\left[A_{j\ell }^{\left(i\right)}=1,A_{j^{\prime }\ell^{\prime }}^{\left(i^{\prime }\right)}=1|s_{1}\right]\mathbb{P}\left[s_{1}\right]+\mathbb{P}\left[A_{j\ell }^{\left(i\right)}=1,A_{j^{\prime }\ell^{\prime }}^{\left(i^{\prime }\right)}=1|s_{2}\right]\mathbb{P}\left[s_{2}\right]\\ =p_{i}p_{i^{\prime }}\Delta_{1}+0\times \Delta_{2}\\ =\Delta_{1}p_{i}p_{i^{\prime }}. \end{array}$$

Note that, because $\Delta_{1}+\Delta_{2}=1$ and $\Phi_{jj^{\prime }}^{\left(1\right)}=\Delta_{2}$ (same with $\Phi_{kk^{\prime }}^{\left(2\right)}$), we have $\Delta_{1}=1-\Phi_{jj^{\prime }}^{\left(1\right)}.$ Thus,

$$\mathbb{P}\left[A_{j\ell }^{\left(i\right)}=1,A_{j^{\prime }\ell^{\prime }}^{\left(i^{\prime }\right)}=1\right]=\left(1-\Phi_{jj^{\prime }}^{\left(1\right)}\right)p_{i}p_{i^{\prime }}$$

and

$$\mathbb{P}\left[B_{kt}^{\left(i\right)}=1,B_{k^{\prime }t^{\prime }}^{\left(i^{\prime }\right)}=1\right]=\left(1-\Phi_{kk^{\prime }}^{\left(2\right)}\right)q_{i}q_{i^{\prime }}.$$

Substituting, we now have

$$E\left[{\overset{˘}{p}}_{i}{\overset{˘}{p}}_{i^{\prime }}{\overset{˘}{q}}_{i}{\overset{˘}{q}}_{i^{\prime }}\right]=\left(\sum_{j=1}^{n_{1}}\sum_{j^{\prime }=1}^{n_{1}}\sum_{\ell =1}^{m_{j}}\sum_{\ell^{\prime }=1}^{m_{j^{\prime }}}\frac{w_{j}w_{j^{\prime }}}{m_{j}m_{j^{\prime }}}\left(1-\Phi_{jj^{\prime }}^{\left(1\right)}\right)p_{i}p_{i^{\prime }}\right)\left(\sum_{k=1}^{n_{2}}\sum_{k^{\prime }=1}^{n_{2}}\sum_{t=1}^{m_{k}}\sum_{t^{\prime }=1}^{m_{k^{\prime }}}\frac{w_{k}w_{k^{\prime }}}{m_{k}m_{k^{\prime }}}\left(1-\Phi_{kk^{\prime }}^{\left(2\right)}\right)q_{i}q_{i^{\prime }}\right)=\left(1-\rho_{2}^{\left(1\right)}\right)\left(1-\rho_{2}^{\left(2\right)}\right)p_{i}p_{i^{\prime }}q_{i}q_{i^{\prime }},$$ (A16)

and substituting Equation A16 into $\text{Cov}\left[{\overset{˘}{p}}_{i}{\overset{˘}{q}}_{i},{\overset{˘}{p}}_{i^{\prime }}{\overset{˘}{q}}_{i^{\prime }}\right]$ yields

$$\begin{array}{c} \text{Cov}\left[{\overset{˘}{p}}_{i}{\overset{˘}{q}}_{i},{\overset{˘}{p}}_{i^{\prime }}{\overset{˘}{q}}_{i^{\prime }}\right]=\left(1-\rho_{2}^{\left(1\right)}\right)\left(1-\rho_{2}^{\left(2\right)}\right)p_{i}p_{i^{\prime }}q_{i}q_{i^{\prime }}-p_{i}q_{i}p_{i^{\prime }}q_{i^{\prime }}.\\ =\left(\rho_{2}^{\left(1\right)}\rho_{2}^{\left(2\right)}-\rho_{2}^{\left(1\right)}-\rho_{2}^{\left(2\right)}\right)p_{i}p_{i^{\prime }}q_{i}q_{i^{\prime }}. \end{array}$$ (A17)

Therefore, using Equations A15 and A17,

$$\text{Var}\left[{\overset{˘}{H}}_{12}\right]=\sum_{i=1}^{I}p_{i}q_{i}\left[\rho_{2}^{\left(1\right)}\left(1-p_{i}\right)q_{i}+\rho_{2}^{\left(2\right)}p_{i}\left(1-q_{i}\right)+\rho_{2}^{\left(1\right)}\rho_{2}^{\left(2\right)}\left(1-p_{i}\right)\left(1-q_{i}\right)\right]+2\sum_{i=1}^{I-1}\sum_{i^{\prime }=i+1}^{I}\left(\rho_{2}^{\left(1\right)}\rho_{2}^{\left(2\right)}-\rho_{2}^{\left(1\right)}-\rho_{2}^{\left(2\right)}\right)p_{i}p_{i^{\prime }}q_{i}q_{i^{\prime }}=\rho_{2}^{\left(1\right)}\sum_{i=1}^{I}p_{i}\left(1-p_{i}\right)q_{i}^{2}+\rho_{2}^{\left(2\right)}\sum_{i=1}^{I}p_{i}^{2}q_{i}\left(1-q_{i}\right)+\rho_{2}^{\left(1\right)}\rho_{2}^{\left(2\right)}\sum_{i=1}^{I}p_{i}\left(1-p_{i}\right)q_{i}\left(1-q_{i}\right)+2\sum_{i=1}^{I-1}\sum_{i^{\prime }=i+1}^{I}\left(\rho_{2}^{\left(1\right)}\rho_{2}^{\left(2\right)}-\rho_{2}^{\left(1\right)}-\rho_{2}^{\left(2\right)}\right)p_{i}p_{i^{\prime }}q_{i}q_{i^{\prime }}=\rho_{2}^{\left(1\right)}\left(1-\rho_{2}^{\left(2\right)}\right)\sum_{i=1}^{I}p_{i}q_{i}^{2}+\rho_{2}^{\left(2\right)}\left(1-\rho_{2}^{\left(1\right)}\right)\sum_{i=1}^{I}p_{i}^{2}q_{i}+\left(\rho_{2}^{\left(1\right)}\rho_{2}^{\left(2\right)}-\rho_{2}^{\left(1\right)}-\rho_{2}^{\left(2\right)}\right)\sum_{i=1}^{I}p_{i}^{2}q_{i}^{2}+\rho_{2}^{\left(1\right)}\rho_{2}^{\left(2\right)}\sum_{i=1}^{I}p_{i}q_{i}+2\left(\rho_{2}^{\left(1\right)}\rho_{2}^{\left(2\right)}-\rho_{2}^{\left(1\right)}-\rho_{2}^{\left(2\right)}\right)\sum_{i=1}^{I}\sum_{i^{\prime }=1}^{I}p_{i}p_{i^{\prime }}q_{i}q_{i^{\prime }}=\rho_{2}^{\left(1\right)}\left(1-\rho_{2}^{\left(2\right)}\right)\sum_{i=1}^{I}p_{i}q_{i}^{2}+\rho_{2}^{\left(2\right)}\left(1-\rho_{2}^{\left(1\right)}\right)\sum_{i=1}^{I}p_{i}^{2}q_{i}+\rho_{2}^{\left(1\right)}\rho_{2}^{\left(2\right)}\sum_{i=1}^{I}p_{i}q_{i}+\left(\rho_{2}^{\left(1\right)}\rho_{2}^{\left(2\right)}-\rho_{2}^{\left(1\right)}-\rho_{2}^{\left(2\right)}\right){\left(\sum_{i=1}^{I}p_{i}q_{i}\right)}^{2}.$$ □

#### Lemma 12:

Consider a locus with *I* distinct alleles across two independent populations and parametric allele frequencies $p_{i}\in \left[0,1\right],$ i=1,2,…,I, and $\sum_{i=1}^{I}p_{i}=1$ for population 1, or $q_{i}\in \left[0,1\right],$ i=1,2,…,I, and $\sum_{i=1}^{I}q_{i}=1$ for population 2. For two samples of size $n_{1}$ and $n_{2}$ individuals from populations 1 and 2, respectively, each with individuals of any ploidy, inbreeding status, and relatedness,

$$\text{Cov}\left[{\overset{˘}{H}}_{12},{\overset{˘}{H}}_{1}\right]=\frac{1}{1-\rho_{2}^{\left(1\right)}}\left[2\left(\rho_{3}^{\left(1\right)}-\rho_{2}^{\left(1\right)}\right)\sum_{i=1}^{I}p_{i}^{3}q_{i}+\left(2\rho_{2}^{\left(1\right)}-3\rho_{3}^{\left(1\right)}\right)\sum_{i=1}^{I}p_{i}^{2}q_{i}+\rho_{3}^{\left(1\right)}\sum_{i=1}^{I}p_{i}q_{i}\right]$$ (A18)

and

$$\text{Cov}\left[{\overset{˘}{H}}_{12},{\overset{˘}{H}}_{2}\right]=\frac{1}{1-\rho_{2}^{\left(2\right)}}\left[2\left(\rho_{3}^{\left(2\right)}-\rho_{2}^{\left(2\right)}\right)\sum_{i=1}^{I}p_{i}q_{i}^{3}+\left(2\rho_{2}^{\left(2\right)}-3\rho_{3}^{\left(2\right)}\right)\sum_{i=1}^{I}p_{i}q_{i}^{2}+\rho_{3}^{\left(2\right)}\sum_{i=1}^{I}p_{i}q_{i}\right],$$ (A19)

where the superscript of the mean kinship coefficients $\rho_{2}$ and $\rho_{3}$ corresponds to the population for which these are calculated. The formulas for $\text{Cov}\left[{\tilde{H}}_{12},{\tilde{H}}_{1}\right],$ $\text{Cov}\left[{\tilde{H}}_{12},{\tilde{H}}_{2}\right],$ $\text{Cov}\left[{\tilde{H}}_{12,\text{BLUE}},{\tilde{H}}_{1,\text{BLUE}}\right],$ and $\text{Cov}\left[{\tilde{H}}_{12,\text{BLUE}},{\tilde{H}}_{2,\text{BLUE}}\right]$ are obtained by substituting ${\bar{\Phi }}_{2}$ and ${\bar{\Phi }}_{3}$ (for $\tilde{H}$), or $\kappa_{2}$ and $\kappa_{3}$ (for ${\tilde{H}}_{\text{BLUE}}$) into Equations A18 and A19, respectively.

#### Proof:

The covariance between ${\overset{˘}{H}}_{12}$ and ${\overset{˘}{H}}_{1}$ is

$$\begin{array}{c} \text{Cov}\left[{\overset{˘}{H}}_{12},{\overset{˘}{H}}_{1}\right]=\frac{1}{1-\rho_{2}^{\left(1\right)}}\text{Cov}\left[\left(1-\sum_{i=1}^{I}{\overset{˘}{p}}_{i}{\overset{˘}{q}}_{i}\right),\left(1-\sum_{i=1}^{I}{\overset{˘}{p}}_{i}^{2}\right)\right]\\ =\frac{1}{1-\rho_{2}^{\left(1\right)}}\sum_{i=1}^{I}\sum_{i^{\prime }=1}^{I}\text{Cov}\left[{\overset{˘}{p}}_{i}{\overset{˘}{q}}_{i},{\overset{˘}{p}}_{i^{\prime }}^{2}\right]\\ =\frac{1}{1-\rho_{2}^{\left(1\right)}}\left(\sum_{i=1}^{I}\text{Cov}\left[{\overset{˘}{p}}_{i}{\overset{˘}{q}}_{i},{\overset{˘}{p}}_{i}^{2}\right]+\sum_{i=1}^{I}\sum_{\begin{array}{l} i^{\prime }=1\\ i^{\prime }\neq i \end{array}}^{I}\text{Cov}\left[{\overset{˘}{p}}_{i}{\overset{˘}{q}}_{i},{\overset{˘}{p}}_{i^{\prime }}^{2}\right]\right). \end{array}$$

The value of the covariance calculated for the case where $i=i^{\prime }$ can be written as

$$\text{Cov}\left[{\overset{˘}{p}}_{i}{\overset{˘}{q}}_{i},{\overset{˘}{p}}_{i}^{2}\right]=E\left[{\overset{˘}{p}}_{i}^{3}{\overset{˘}{q}}_{i}\right]-E\left[{\overset{˘}{p}}_{i}{\overset{˘}{q}}_{i}\right]E\left[{\overset{˘}{p}}_{i}^{2}\right].$$

From the proof of Lemma 11, we have derived the value of $E\left[{\overset{˘}{p}}_{i}{\overset{˘}{q}}_{i}\right],$ and, from the proof of Proposition 1 we know the value for $E\left[{\overset{˘}{p}}_{i}^{2}\right].$ We therefore only need to compute

$$E\left[{\overset{˘}{p}}_{i}^{3}{\overset{˘}{q}}_{i}\right]=E\left[{\overset{˘}{p}}_{i}^{3}\right]E\left[{\overset{˘}{q}}_{i}\right],$$

where $E\left[{\overset{˘}{q}}_{i}\right]=q_{i}.$ Solving for $E\left[{\overset{˘}{p}}_{i}^{3}\right],$ we have

$$\begin{array}{c} E\left[{\overset{˘}{p}}_{i}^{3}\right]=\sum_{j=1}^{n_{1}}\sum_{v=1}^{n_{1}}\sum_{v^{\prime }=1}^{n_{1}}\sum_{\ell =1}^{m_{j}}\sum_{z=1}^{m_{v}}\sum_{z^{\prime }=1}^{m_{v^{\prime }}}\frac{w_{j}w_{v}w_{v^{\prime }}}{m_{j}m_{v}m_{v^{\prime }}}E\left[A_{j\ell }^{\left(i\right)}A_{vz}^{\left(i\right)}A_{v^{\prime }z^{\prime }}^{\left(i\right)}\right]\\ =\sum_{j=1}^{n_{1}}\sum_{v=1}^{n_{1}}\sum_{v^{\prime }=1}^{n_{1}}\sum_{\ell =1}^{m_{j}}\sum_{z=1}^{m_{v}}\sum_{z^{\prime }=1}^{m_{v^{\prime }}}\frac{w_{j}w_{v}w_{v^{\prime }}}{m_{j}m_{v}m_{v^{\prime }}}\mathbb{P}\left[A_{j\ell }^{\left(i\right)}=1,A_{vz}^{\left(i\right)}=1,A_{v^{\prime }z^{\prime }}^{\left(i\right)}=1\right]. \end{array}$$

The value of $\mathbb{P}\left[A_{j\ell }^{\left(i\right)}=1,A_{vz}^{\left(i\right)}=1,A_{v\prime z\prime }^{\left(i\right)}=1\right]$ depends on the probabilities of distinct identity states in which three alleles are drawn from the sample (one each from individuals *j*, *v*, and $v^{\prime }$). We define state 1 as no IBD alleles drawn (probability $\delta_{1}$), state 2 as IBD alleles drawn from *j* and *v* (probability $\delta_{2}$), state 3 as IBD alleles drawn from *v* and $v^{\prime }$ IBD (probability $\delta_{3}$), state 4 as IBD alleles drawn from *j* and $v^{\prime }$ IBD (probability $\delta_{4}$), and state 5 as all three IBD (probability $\delta_{5}$), with $\sum_{s=1}^{5}\delta_{s}=1.$ Thus, the probabilities for the relevant kinship coefficients are

$$\Phi_{jvv^{\prime }}^{\left(1\right)}=\delta_{5}$$

$$\Phi_{jv}^{\left(1\right)}=\delta_{5}+\delta_{2}$$

$$\Phi_{vv^{\prime }}^{\left(1\right)}=\delta_{5}+\delta_{3}$$

$$\Phi_{jv^{\prime }}^{\left(1\right)}=\delta_{5}+\delta_{4},$$

which yields

$$\begin{array}{l} \mathbb{P}\left[A_{j\ell }^{\left(i\right)}=1,A_{vz}^{\left(i\right)}=1,A_{v^{\prime }z^{\prime }}^{\left(i\right)}=1\right]=\delta_{5}p_{i}+\left(\delta_{2}+\delta_{3}+\delta_{4}\right)p_{i}^{2}+\delta_{1}p_{i}^{3}\\ \;=\Phi_{jvv^{\prime }}^{\left(1\right)}p_{i}+\left(\Phi_{jv}^{\left(1\right)}+\Phi_{vv^{\prime }}^{\left(1\right)}+\Phi_{jv^{\prime }}^{\left(1\right)}-3\Phi_{jvv^{\prime }}^{\left(1\right)}\right)p_{i}^{2}+\left(1+2\Phi_{jvv^{\prime }}^{\left(1\right)}-\Phi_{jv^{\prime }}^{\left(1\right)}-\Phi_{vv^{\prime }}^{\left(1\right)}-\Phi_{jv}^{\left(1\right)}\right)p_{i}^{3}. \end{array}$$

Thus, $E\left[{\overset{˘}{p}}_{i}^{3}{\overset{˘}{q}}_{i}\right]$ is

$$E\left[{\overset{˘}{p}}_{i}^{3}{\overset{˘}{q}}_{i}\right]=\rho_{3}^{\left(1\right)}p_{i}q_{i}+3\left(\rho_{2}^{\left(1\right)}-\rho_{3}^{\left(1\right)}\right)p_{i}^{2}q_{i}+\left(1+2\rho_{3}^{\left(1\right)}-3\rho_{2}^{\left(1\right)}\right)p_{i}^{3}q_{i},$$ (A20)

and from Equations A20 and A1, and the definition of $E\left[{\overset{˘}{p}}_{i}{\overset{˘}{q}}_{i}\right],$

$$\begin{array}{c} \text{Cov}\left[{\overset{˘}{p}}_{i}{\overset{˘}{q}}_{i},{\overset{˘}{p}}_{i}^{2}\right]=\rho_{3}^{\left(1\right)}p_{i}q_{i}+3\left(\rho_{2}^{\left(1\right)}-\rho_{3}^{\left(1\right)}\right)p_{i}^{2}q_{i}+\left(1+2\rho_{3}^{\left(1\right)}-3\rho_{2}^{\left(1\right)}\right)p_{i}^{3}q_{i}-p_{i}q_{i}\left[p_{i}^{2}+\rho_{2}^{\left(1\right)}p_{i}\left(1-p_{i}\right)\right]\\ =2\left(\rho_{3}^{\left(1\right)}-\rho_{2}^{\left(1\right)}\right)p_{i}^{3}q_{i}+\left(2\rho_{2}^{\left(1\right)}-3\rho_{3}^{\left(1\right)}\right)p_{i}^{2}q_{i}+\rho_{3}^{\left(1\right)}p_{i}q_{i}. \end{array}$$ (A21)

Meanwhile, for the $\text{Cov}\left[{\overset{˘}{p}}_{i}{\overset{˘}{q}}_{i},{\overset{˘}{p}}_{i^{\prime }}^{2}\right]$ case of $i\neq i^{\prime },$ $\text{Cov}\left[{\overset{˘}{p}}_{i}{\overset{˘}{q}}_{i},{\overset{˘}{p}}_{i^{\prime }}^{2}\right]=0.$ This is intuitively sensible because the products ${\overset{˘}{p}}_{i}{\overset{˘}{q}}_{i}$ and ${\overset{˘}{p}}_{i^{\prime }}^{2}$ are independent, describing different alleles, and should not covary.

Finally, we can see that, when the two populations considered are independent from one another, the value of $\text{Cov}\left[{\overset{˘}{H}}_{12},{\overset{˘}{H}}_{1}\right]$ (or equivalently of $\text{Cov}\left[{\overset{˘}{H}}_{12},{\overset{˘}{H}}_{2}\right]$) is driven entirely by the case in which $i=i^{\prime },$ such that

$$\begin{array}{c} \mathrm{Cov}\left[{\overset{˘}{H}}_{12},{\overset{˘}{H}}_{1}\right]=\frac{1}{1-\rho_{2}^{\left(1\right)}}\sum_{i=1}^{I}\text{Cov}\left[{\overset{˘}{p}}_{i}{\overset{˘}{q}}_{i},{\overset{˘}{p}}_{i}^{2}\right]\\ =\frac{1}{1-\rho_{2}^{\left(1\right)}}\left[2\left(\rho_{3}^{\left(1\right)}-\rho_{2}^{\left(1\right)}\right)\sum_{i=1}^{I}p_{i}^{3}q_{i}+\left(2\rho_{2}^{\left(1\right)}-3\rho_{3}^{\left(1\right)}\right)\sum_{i=1}^{I}p_{i}^{2}q_{i}+\rho_{3}^{\left(1\right)}\sum_{i=1}^{I}p_{i}q_{i}\right]. \end{array}$$ □

We now need to derive $\text{Cov}\left[{\overset{˘}{H}}_{12}-\frac{1}{2}\left({\overset{˘}{H}}_{1}+{\overset{˘}{H}}_{2}\right),{\overset{˘}{H}}_{12}\right],$ the final term required to compute $\mathrm{Var}\left[{\overset{˘}{F}}_{\text{ST}}\right].$

#### Lemma 13:

Consider a locus with *I* distinct alleles across two independent populations and parametric allele frequencies $p_{i}\in \left[0,1\right],$ i=1,2,…,I, and $\sum_{i=1}^{I}p_{i}=1$ for population 1, or $q_{i}\in \left[0,1\right],$ i=1,2,…,I, and $\sum_{i=1}^{I}q_{i}=1$ for population 2. For two samples of size $n_{1}$ and $n_{2}$ individuals from populations 1 and 2, respectively, each with individuals of any ploidy, inbreeding status, and relatedness,

$$\begin{array}{c} \text{Cov}\left[{\overset{˘}{H}}_{12}-\frac{1}{2}{\overset{˘}{H}}_{1}-\frac{1}{2}{\overset{˘}{H}}_{2},{\overset{˘}{H}}_{12}\right]=\left[\rho_{2}^{\left(1\right)}\left(1-\rho_{2}^{\left(2\right)}\right)+\frac{3\rho_{3}^{\left(2\right)}-2\rho_{2}^{\left(2\right)}}{2\left(1-\rho_{2}^{\left(2\right)}\right)}\right]\sum_{i=1}^{I}p_{i}q_{i}^{2}+\left[\rho_{2}^{\left(2\right)}\left(1-\rho_{2}^{\left(1\right)}\right)+\frac{3\rho_{3}^{\left(1\right)}-2\rho_{2}^{\left(1\right)}}{2\left(1-\rho_{2}^{\left(1\right)}\right)}\right]\overset{I}{\underset{i=1}{\sum }}p_{i}^{2}q_{i}\\ +\left[\rho_{2}^{\left(1\right)}\rho_{2}^{\left(2\right)}-\frac{\rho_{3}^{\left(1\right)}}{2\left(1-\rho_{2}^{\left(1\right)}\right)}-\frac{\rho_{3}^{\left(2\right)}}{2\left(1-\rho_{2}^{\left(2\right)}\right)}\right]\sum_{i=1}^{I}p_{i}q_{i}+\left(\rho_{2}^{\left(1\right)}\rho_{2}^{\left(2\right)}-\rho_{2}^{\left(1\right)}-\rho_{2}^{\left(2\right)}\right){\left(\sum_{i=1}^{I}p_{i}q_{i}\right)}^{2}\\ +\frac{\rho_{2}^{\left(1\right)}-\rho_{3}^{\left(1\right)}}{1-\rho_{2}^{\left(1\right)}}\sum_{i=1}^{I}p_{i}^{3}q_{i}+\frac{\rho_{2}^{\left(2\right)}-\rho_{3}^{\left(2\right)}}{1-\rho_{2}^{\left(2\right)}}\sum_{i=1}^{I}p_{i}q_{i}^{3}, \end{array}$$ (A22)

where the superscript of the mean kinship coefficients $\rho_{2}$ and $\rho_{3}$ corresponds to the population for which these quantities are calculated. The formulas for $\text{Cov}\left[{\tilde{H}}_{12}-\left(1/2\right){\tilde{H}}_{1}-\left(1/2\right){\tilde{H}}_{2},{\tilde{H}}_{12}\right]$ and $\mathrm{Cov}\left[{\tilde{H}}_{12,\text{BLUE}}-\left(1/2\right){\tilde{H}}_{1,\text{BLUE}}-\left(1/2\right){\tilde{H}}_{2,\text{BLUE}},{\tilde{H}}_{12,\text{BLUE}}\right]$ are obtained by substituting ${\bar{\Phi }}_{2}$ and ${\bar{\Phi }}_{3}$ (for $\tilde{H}$), or $\kappa_{2}$ and $\kappa_{3}$ (for ${\tilde{H}}_{\text{BLUE}}$) into Equation A22.

#### Proof:

We begin by breaking up the covariance into its components,

$$\mathrm{Cov}\left[{\overset{˘}{H}}_{12}-\frac{1}{2}{\overset{˘}{H}}_{1}-\frac{1}{2}{\overset{˘}{H}}_{2},{\overset{˘}{H}}_{12}\right]=\mathrm{Var}\left[{\overset{˘}{H}}_{12}\right]-\frac{1}{2}\mathrm{Cov}\left[{\overset{˘}{H}}_{1},{\overset{˘}{H}}_{12}\right]-\frac{1}{2}\mathrm{Cov}\left[{\overset{˘}{H}}_{2},{\overset{˘}{H}}_{12}\right].$$

This equation is composed of terms that we previously derived (Equations A13, A18, and A19). Therefore,

$$\begin{array}{c} \text{Cov}\left[{\overset{˘}{H}}_{12}-\frac{1}{2}{\overset{˘}{H}}_{1}-\frac{1}{2}{\overset{˘}{H}}_{2},{\overset{˘}{H}}_{12}\right]=\rho_{2}^{\left(1\right)}\left(1-\rho_{2}^{\left(2\right)}\right)\sum_{i=1}^{I}p_{i}q_{i}^{2}+\rho_{2}^{\left(2\right)}\left(1-\rho_{2}^{\left(1\right)}\right)\sum_{i=1}^{I}p_{i}^{2}q_{i}+\rho_{2}^{\left(1\right)}\rho_{2}^{\left(2\right)}\sum_{i=1}^{I}p_{i}q_{i}\\ +\left(\rho_{2}^{\left(1\right)}\rho_{2}^{\left(2\right)}-\rho_{2}^{\left(1\right)}-\rho_{2}^{\left(2\right)}\right){\left(\sum_{i=1}^{I}p_{i}q_{i}\right)}^{2}\\ -\frac{1}{2\left(1-\rho_{2}^{\left(1\right)}\right)}\left[2\left(\rho_{3}^{\left(1\right)}-\rho_{2}^{\left(1\right)}\right)\sum_{i=1}^{I}p_{i}^{3}q_{i}+\left(2\rho_{2}^{\left(1\right)}-3\rho_{3}^{\left(1\right)}\right)\sum_{i=1}^{I}p_{i}^{2}q_{i}+\rho_{3}^{\left(1\right)}\sum_{i=1}^{I}p_{i}q_{i}\right]\\ -\frac{1}{2\left(1-\rho_{2}^{\left(2\right)}\right)}\left[2\left(\rho_{3}^{\left(2\right)}-\rho_{2}^{\left(2\right)}\right)\sum_{i=1}^{I}p_{i}q_{i}^{3}+\left(2\rho_{2}^{\left(2\right)}-3\rho_{3}^{\left(2\right)}\right)\sum_{i=1}^{I}p_{i}q_{i}^{2}+\rho_{3}^{\left(2\right)}\sum_{i=1}^{I}p_{i}q_{i}\right]\\ =\left[\rho_{2}^{\left(1\right)}\left(1-\rho_{2}^{\left(2\right)}\right)+\frac{3\rho_{3}^{\left(2\right)}-2\rho_{2}^{\left(2\right)}}{2\left(1-\rho_{2}^{\left(2\right)}\right)}\right]\sum_{i=1}^{I}p_{i}q_{i}^{2}+\left[\rho_{2}^{\left(2\right)}\left(1-\rho_{2}^{\left(1\right)}\right)+\frac{3\rho_{3}^{\left(1\right)}-2\rho_{2}^{\left(1\right)}}{2\left(1-\rho_{2}^{\left(1\right)}\right)}\right]\sum_{i=1}^{I}p_{i}^{2}q_{i}\\ +\left[\rho_{2}^{\left(1\right)}\rho_{2}^{\left(2\right)}-\frac{\rho_{3}^{\left(1\right)}}{2\left(1-\rho_{2}^{\left(1\right)}\right)}-\frac{\rho_{3}^{\left(2\right)}}{2\left(1-\rho_{2}^{\left(2\right)}\right)}\right]\sum_{i=1}^{I}p_{i}q_{i}+\left(\rho_{2}^{\left(1\right)}\rho_{2}^{\left(2\right)}-\rho_{2}^{\left(1\right)}-\rho_{2}^{\left(2\right)}\right){\left(\sum_{i=1}^{I}p_{i}q_{i}\right)}^{2}\\ +\frac{\rho_{2}^{\left(1\right)}-\rho_{3}^{\left(1\right)}}{1-\rho_{2}^{\left(1\right)}}\sum_{i=1}^{I}p_{i}^{3}q_{i}+\frac{\rho_{2}^{\left(2\right)}-\rho_{3}^{\left(2\right)}}{1-\rho_{2}^{\left(2\right)}}\sum_{i=1}^{I}p_{i}q_{i}^{3}. \end{array}$$ □
