# Supplementary material for: An Unbiased Estimator of Gene Diversity with Improved Variance for Samples Containing Related and Inbred Individuals of any Ploidy
Source: G3 (Bethesda). 2016 Dec 30;7(2):671–91. doi: 10.1534/g3.116.037168 (PMC5295611; doi:10.1534/g3.116.037168)
Supplement: Supplementary file 1 [file 671FigureS1.pdf]

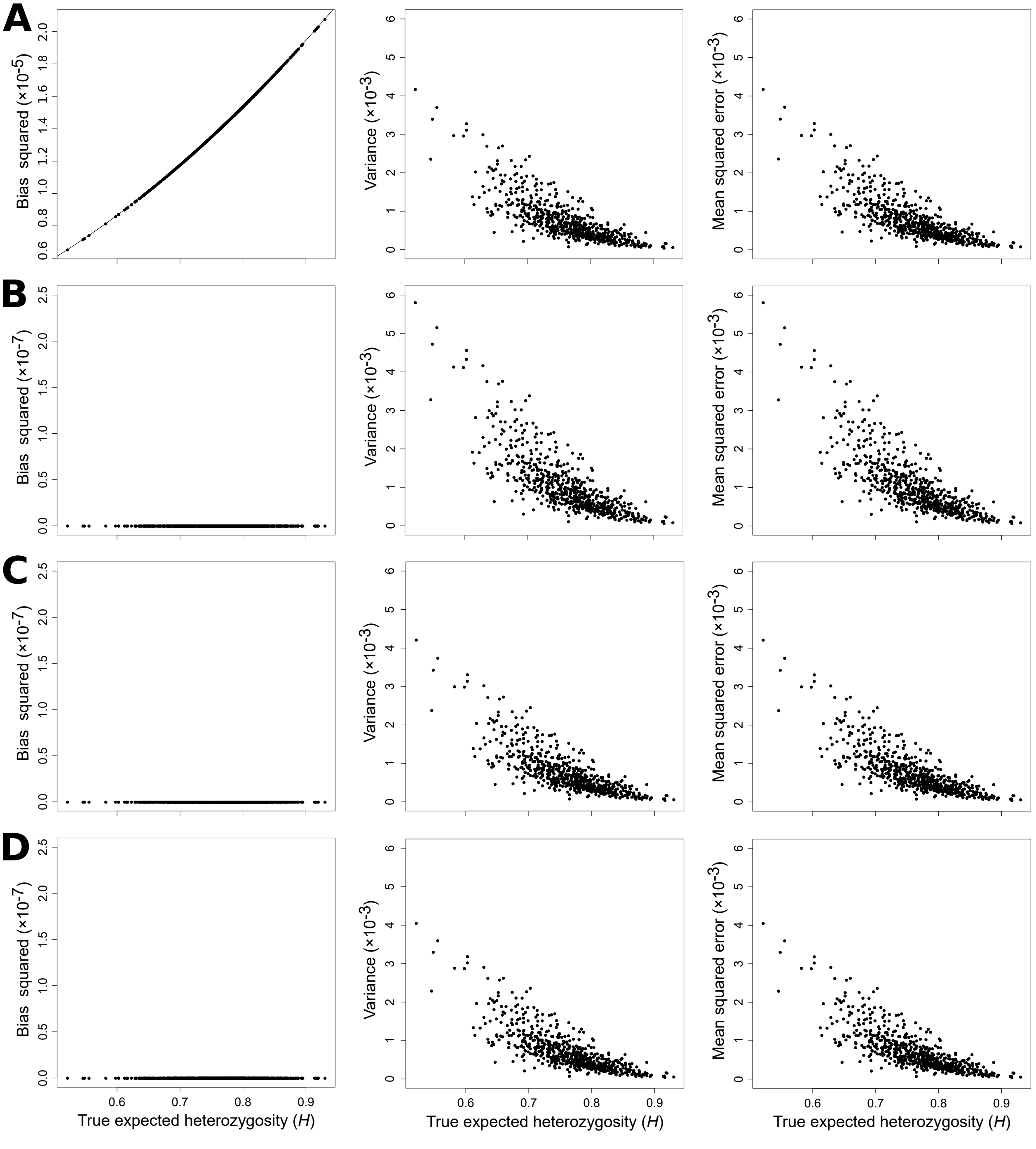


Figure S1: Theoretical squared bias, variance, and mean squared error of the estimators $\widehat{H}_{\text{full}}$ (A), $\widehat{H}_{\text{red}}$ (B), $\widetilde{H}$ (C), and $\widetilde{H}_{\text{BLUE}}$ (D), calculated at each of 645 loci ($0.5212 \leq H \leq 0.9301$) in the MS5795 dataset for 60 individuals wherein each individual was related to exactly one other. The relatedness of the sample was fixed at 10 inbred full-siblings ($\Phi = 3/8$), 10 outbred full-siblings ($\Phi = 1/4$), and 10 outbred avuncular pairs ($\Phi = 1/8$). The curve in the squared bias panel of (A) is described in Equation A10. See Supplementary Data for the true expected heterozygosity values incorporated into those calculations.
