# Supplementary material for: An Unbiased Estimator of Gene Diversity with Improved Variance for Samples Containing Related and Inbred Individuals of any Ploidy
Source: G3 (Bethesda). 2016 Dec 30;7(2):671–91. doi: 10.1534/g3.116.037168 (PMC5295611; doi:10.1534/g3.116.037168)
Supplement: Supplementary file 2 [file 671FigureS2.pdf]

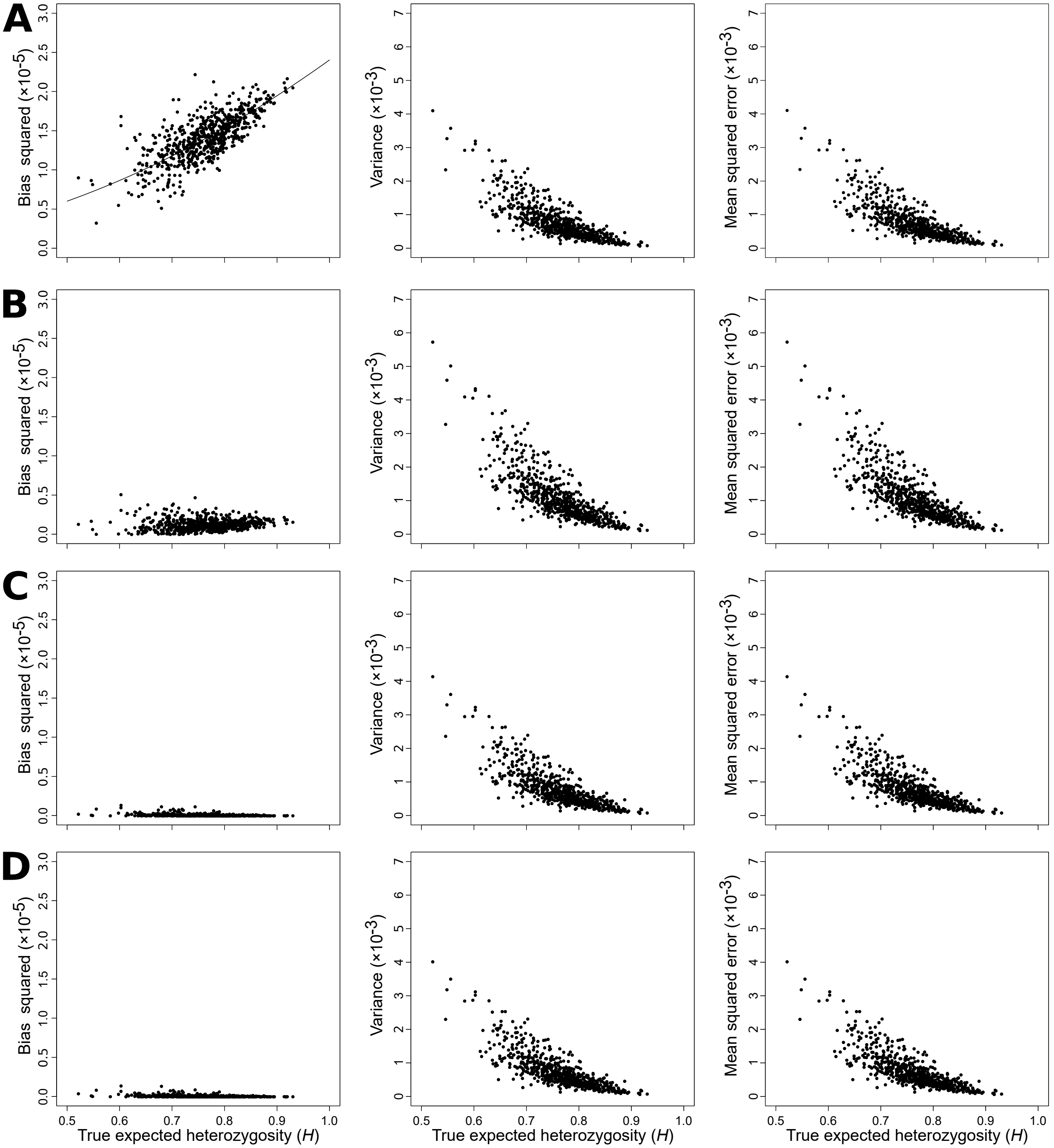

Figure S2: Squared bias, variance, and mean squared error of the estimators $\widehat{H}_{\text{full}}$ (A), $\widehat{H}_{\text{red}}$ (B), $\widetilde{H}$ (C), and $\widetilde{H}_{\text{BLUE}}$ (D), obtained from simulated values at each of 645 loci ($0.5212 \leq H \leq 0.9301$) in the MS5795 dataset for 60 individuals wherein each individual was related to exactly one other. The relatedness of the sample was fixed at 10 inbred full-siblings ($\Phi = 3/8$), 10 outbred full-siblings ($\Phi = 1/4$), and 10 outbred avuncular pairs ($\Phi = 1/8$). Each point in the graphs is based on the mean of 10^4 simulations. The relatedness of the sample was fixed at 10 inbred full-siblings, 10 outbred full-siblings, and 10 outbred avuncular pairs. The curve in the squared bias panel of (A) is described in Equation A10. See Supplementary Data for the true expected heterozygosity values incorporated into the calculation of the curve.
