# Supplementary material for: An Unbiased Estimator of Gene Diversity with Improved Variance for Samples Containing Related and Inbred Individuals of any Ploidy
Source: G3 (Bethesda). 2016 Dec 30;7(2):671–91. doi: 10.1534/g3.116.037168 (PMC5295611; doi:10.1534/g3.116.037168)
Supplement: Supplementary file 3 [file 671FigureS3.pdf]

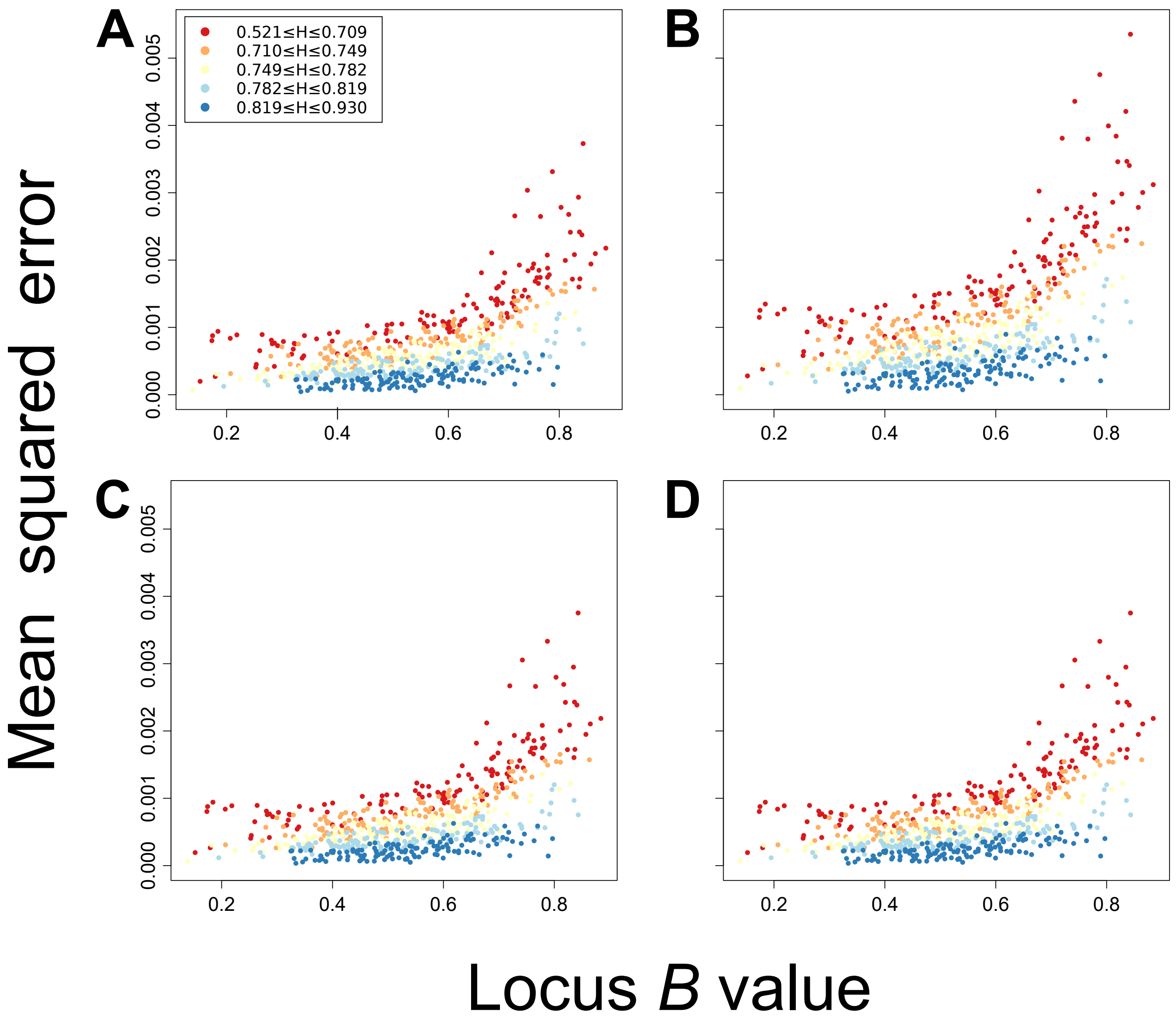

Figure S3: Theoretical mean squared error of the estimators (A) $\widehat{H}_{\text{full}}$, (B) $\widehat{H}_{\text{red}}$, (C) $\widetilde{H}$, (D) and $\widetilde{H}_{\text{BLUE}}$ for the MS5795 dataset with the horizontal axis representing a quantity we call B in the *Results*. B takes on values between 0 and 1 and quantifies the gene diversity at a locus as a proportion of the range of H values available to the locus, given its associated number of alleles (I) and the frequency of the most frequent allele (M). The minimum value of H for a locus is defined as $H_{\text{min}} = M\left(\lceil M^{-1}\rceil - 1\right)\left(2 - \lceil M^{-1}\rceil M\right)$, while the maximum value is $H_{\text{max}} = 1 - (IM^2 - 2M + 1)/(I-1)$. Thus, $B = D/R$, where $D = H - H_{\text{min}}$ and $R = H_{\text{max}} - H_{\text{min}}$. The 645 locus values $(0.5212 \leq H \leq 0.9301)$ are colored by quintile in order of increasing H, with red representing the quintile with the lowest gene diversity and blue representing the quintile with the highest. Note that the order in which loci appear as points on the horizontal axis is not related to quintile as it is in Supplementary Figure S1 (where the horizontal axis measures H).
