# Supplementary material for: An Unbiased Estimator of Gene Diversity with Improved Variance for Samples Containing Related and Inbred Individuals of any Ploidy
Source: G3 (Bethesda). 2016 Dec 30;7(2):671–91. doi: 10.1534/g3.116.037168 (PMC5295611; doi:10.1534/g3.116.037168)
Supplement: Supplementary file 5 [file 671FigureS5.pdf]

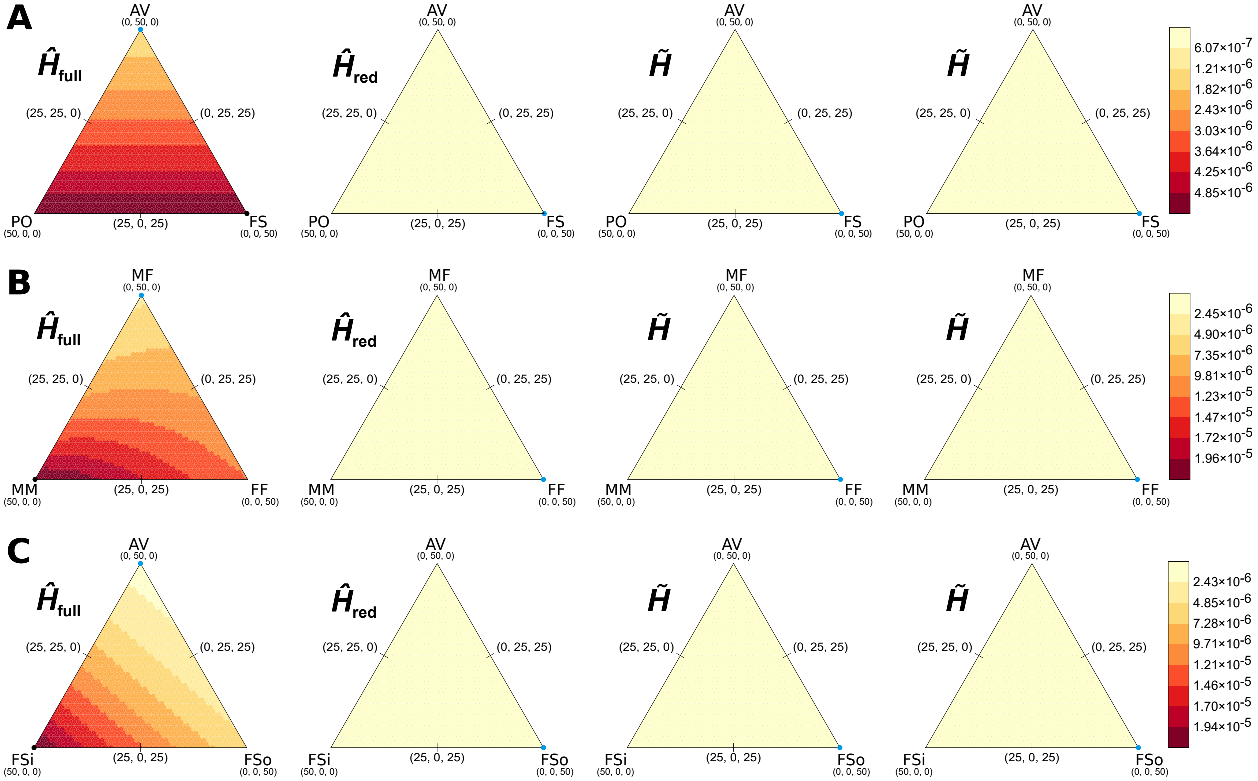

Figure S5: Theoretical squared bias of the estimators $\widehat{H}_{\text{full}}$, $\widehat{H}_{\text{red}}$, $\widetilde{H}$, and $\widetilde{H}_{\text{BLUE}}$ for samples fixed at 100 (A) diploid individuals, (B) male and female individuals at an X-linked locus, or (C) diploid individuals wherein some full siblings are inbred with brother-sister parents, across changing sample configurations. The samples were modeled on the D3S2427 locus and contained 50 relative pairs: (A) parent-offspring (PO), second-degree avuncular (AV), and full-sibling (FS), (B) male-male (MM), male-female (MF), and female-female (FF) full-sibling, or (C) inbred full-sibling (FSi), second-degree avuncular (AV), and outbred full-sibling (FSo), such that each individual was related to exactly one other. The vertices of the heat map represent samples composed of only one relative pair type while the space between them corresponds to all possible combinations of relative pair types. Blue and black points indicate the smallest and largest values on the map, respectively. The heat maps of unbiased estimators (second, third and fourth triangles) contain only a blue point because the value of the squared bias is zero throughout the space of the sample configurations. Threshold values for coloration are indicated in the scales to the right of the figure, with smaller values colored lighter.
