# Supplementary material for: An Unbiased Estimator of Gene Diversity with Improved Variance for Samples Containing Related and Inbred Individuals of any Ploidy
Source: G3 (Bethesda). 2016 Dec 30;7(2):671–91. doi: 10.1534/g3.116.037168 (PMC5295611; doi:10.1534/g3.116.037168)
Supplement: Supplementary file 7 [file 671FigureS7.pdf]

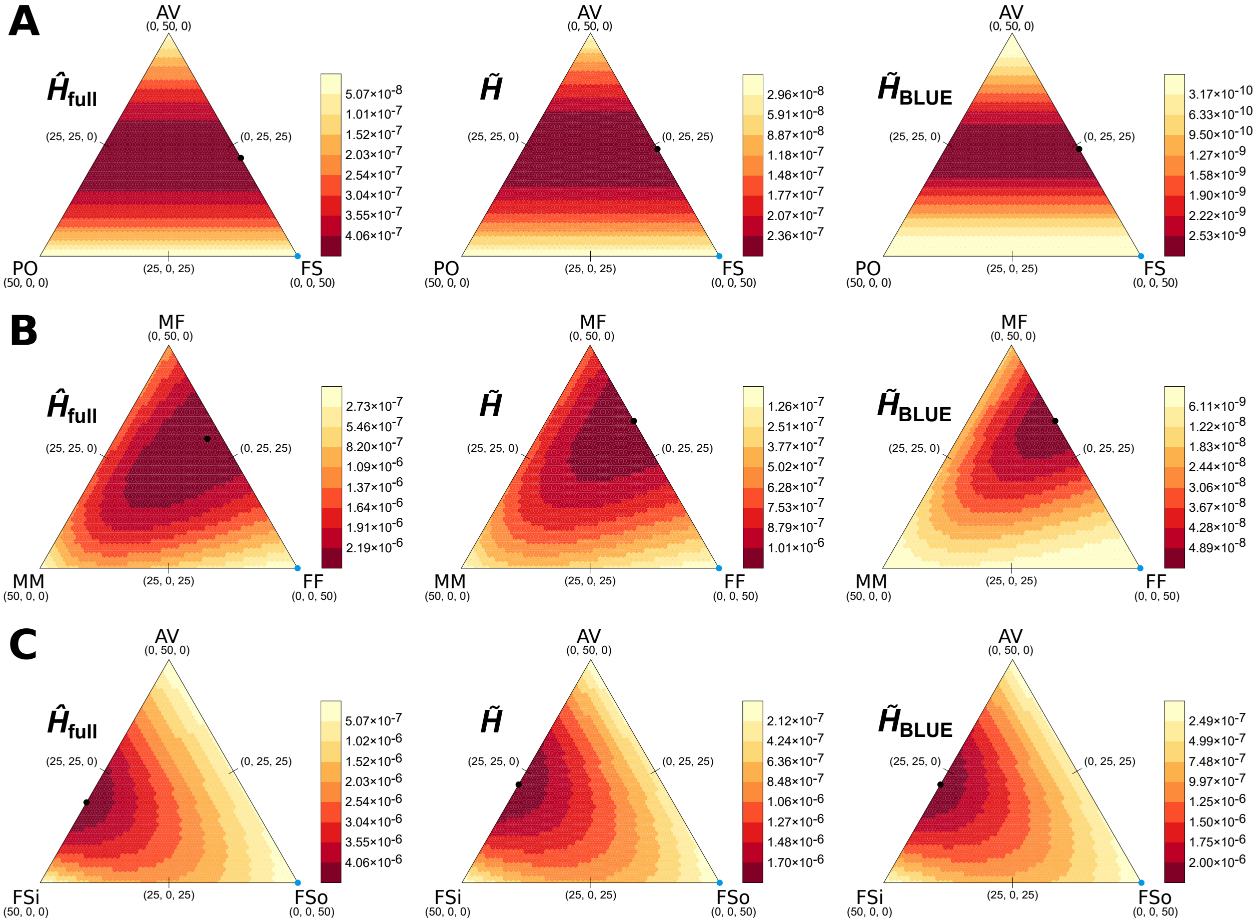

Figure S7: Theoretical difference in mean squared error between the estimators $\widehat{H}_{\text{full}}$, $\widehat{H}_{\text{red}}$, $\widetilde{H}$, and $\widetilde{H}_{\text{BLUE}}$ for samples fixed at 100 (A) diploid individuals, (B) male and female individuals at an X-linked locus, or (C) diploid individuals wherein some full siblings are inbred with brother-sister parents, across changing sample configurations. Here, the MSE of the estimator calculated with $\widetilde{p}_i$ is subtracted from that of the same estimator calculated with $\widehat{p}_i$, yielding a value greater than zero. The samples considered for each subtraction are as in Supplementary Figures S4-S6. Threshold values for coloration are indicated in the scales to the right of each triangle. Note that scales are not identical across heat maps. A heat map corresponding to $\widehat{H}_{\text{red}}$ is not included because all sample configurations yield a value of zero for this calculation.
