# Supplementary figures and images for: An Unbiased Estimator of Gene Diversity with Improved Variance for Samples Containing Related and Inbred Individuals of any Ploidy
Source: G3 (Bethesda). 2016 Dec 30;7(2):671–91. doi: 10.1534/g3.116.037168 (PMC5295611; doi:10.1534/g3.116.037168)

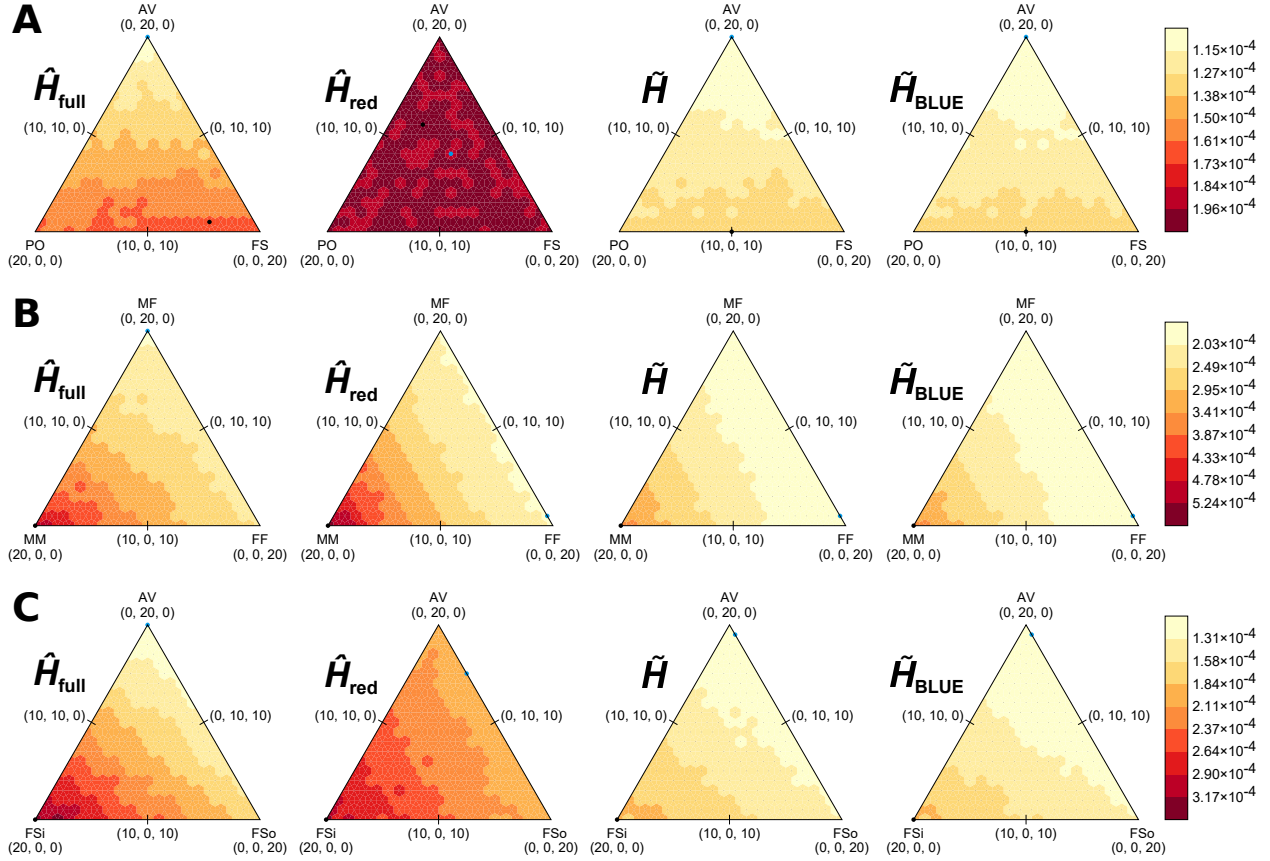

Supplement: Supplementary file 8 [file 671FigureS8.pdf]
