# Supplementary material for: An Unbiased Estimator of Gene Diversity with Improved Variance for Samples Containing Related and Inbred Individuals of any Ploidy
Source: G3 (Bethesda). 2016 Dec 30;7(2):671–91. doi: 10.1534/g3.116.037168 (PMC5295611; doi:10.1534/g3.116.037168)
Supplement: Supplementary file 9 [file 671FigureS9.pdf]

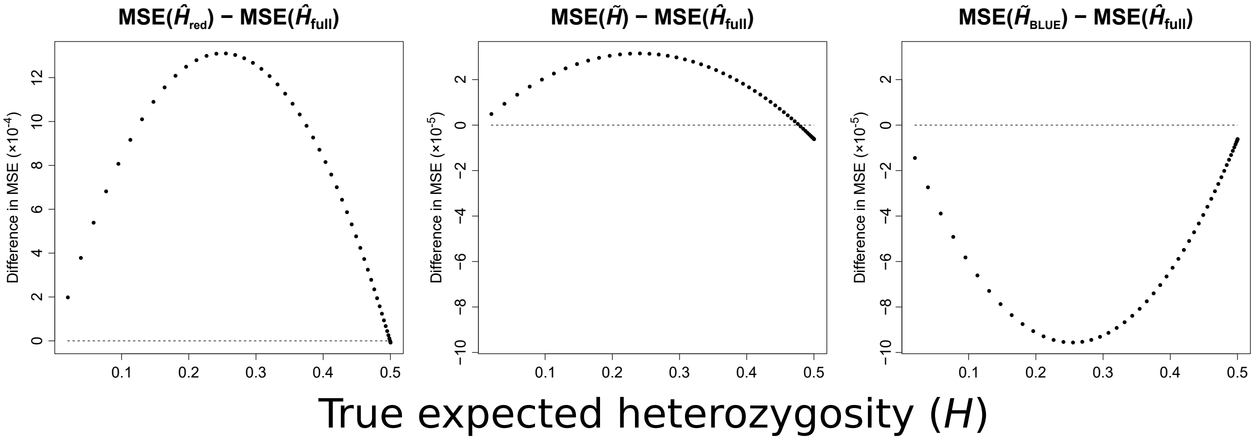

Figure S9: Theoretical difference in mean squared error between the unbiased estimator $\widehat{H}_{\text{red}}$ (left), $\widetilde{H}$ (center), or $\widetilde{H}_{\text{BLUE}}$ (right) and the biased estimator $\widehat{H}_{\text{full}}$ calculated at each of 50 SNP simulated loci ($0.0198 \le H \le 0.5$) for samples of 60 diploid individuals containing some inbred relative pairs. Each sampled individual was related to exactly one other, and samples contained 10 pairs of inbred full-siblings ($\Phi = 3/8$), 10 pairs of outbred full-siblings ($\Phi = 1/4$), and 10 outbred avuncular pairs ($\Phi = 1/8$). Dotted lines in each plot correspond to a difference in MSE of zero with $\widehat{H}_{\text{full}}$. See Supplementary Data for the true expected heterozygosity values incorporated into analytical calculations (SNP loci are labeled as SNP01-SNP50).
