# Supplementary material for: An Unbiased Estimator of Gene Diversity with Improved Variance for Samples Containing Related and Inbred Individuals of any Ploidy
Source: G3 (Bethesda). 2016 Dec 30;7(2):671–91. doi: 10.1534/g3.116.037168 (PMC5295611; doi:10.1534/g3.116.037168)
Supplement: Supplementary file 10 [file 671FigureS10.pdf]

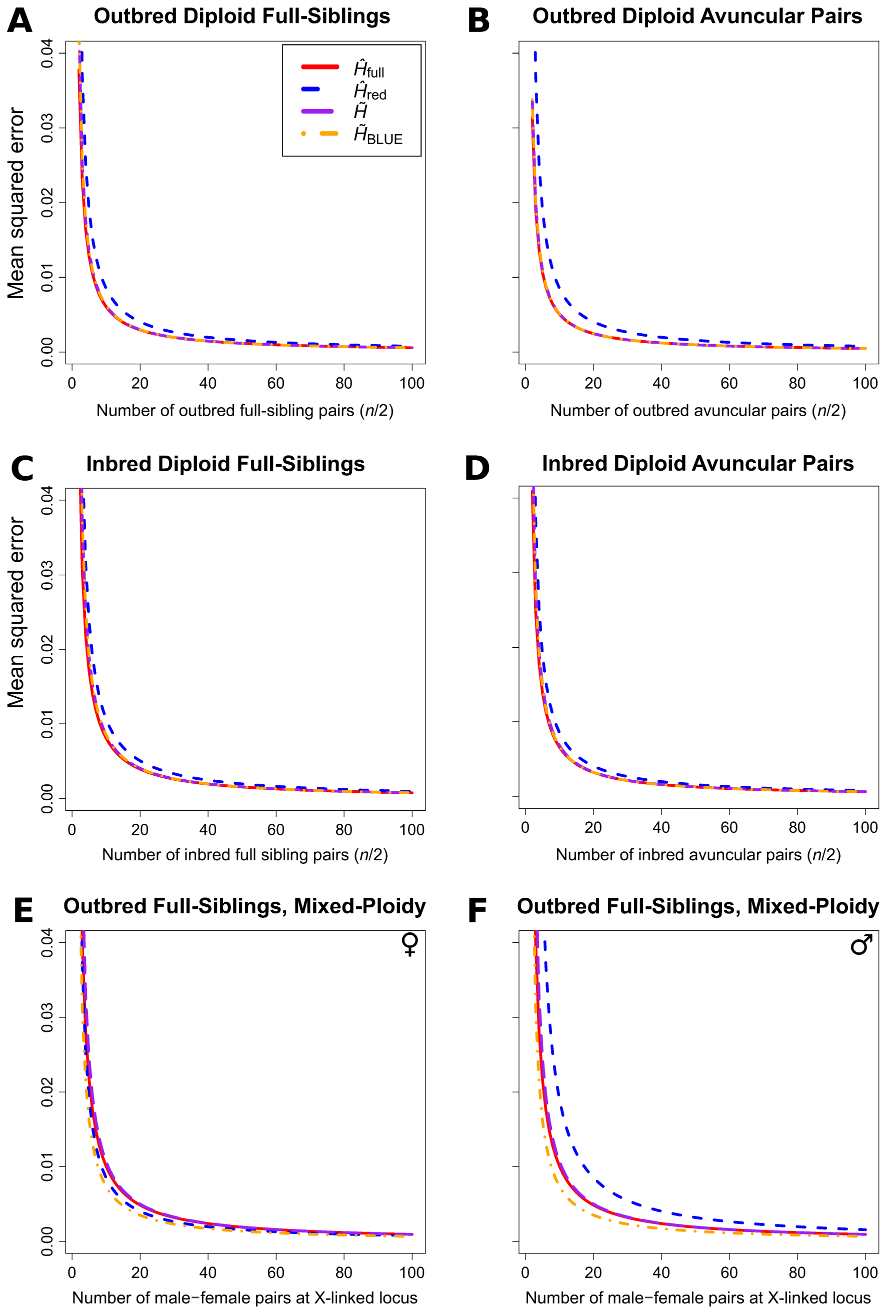


Figure S10: Theoretical mean squared error as a function of sample size for samples of outbred diploid full-siblings (A), outbred diploid avuncular pairs (B), inbred diploid full-siblings (C), inbred diploid avuncular pairs (D), male-female full siblings at an X-linked locus with the reduced set omitting males and retaining females (E), and male-female full siblings at an X-linked locus with the reduced set omitting females and retaining males (F). The samples were evaluated for a biallelic locus with minor allele frequency of 0.05 ($H = 0.095$), and sample size was always twice the number of relative pairs included in the sample for samples containing 2 to 100 relative pairs. Each individual in the sample was related to exactly one other.
