# Supplementary material for: An Unbiased Estimator of Gene Diversity with Improved Variance for Samples Containing Related and Inbred Individuals of any Ploidy
Source: G3 (Bethesda). 2016 Dec 30;7(2):671–91. doi: 10.1534/g3.116.037168 (PMC5295611; doi:10.1534/g3.116.037168)
Supplement: Supplementary file 11 [file 671FigureS11.pdf]

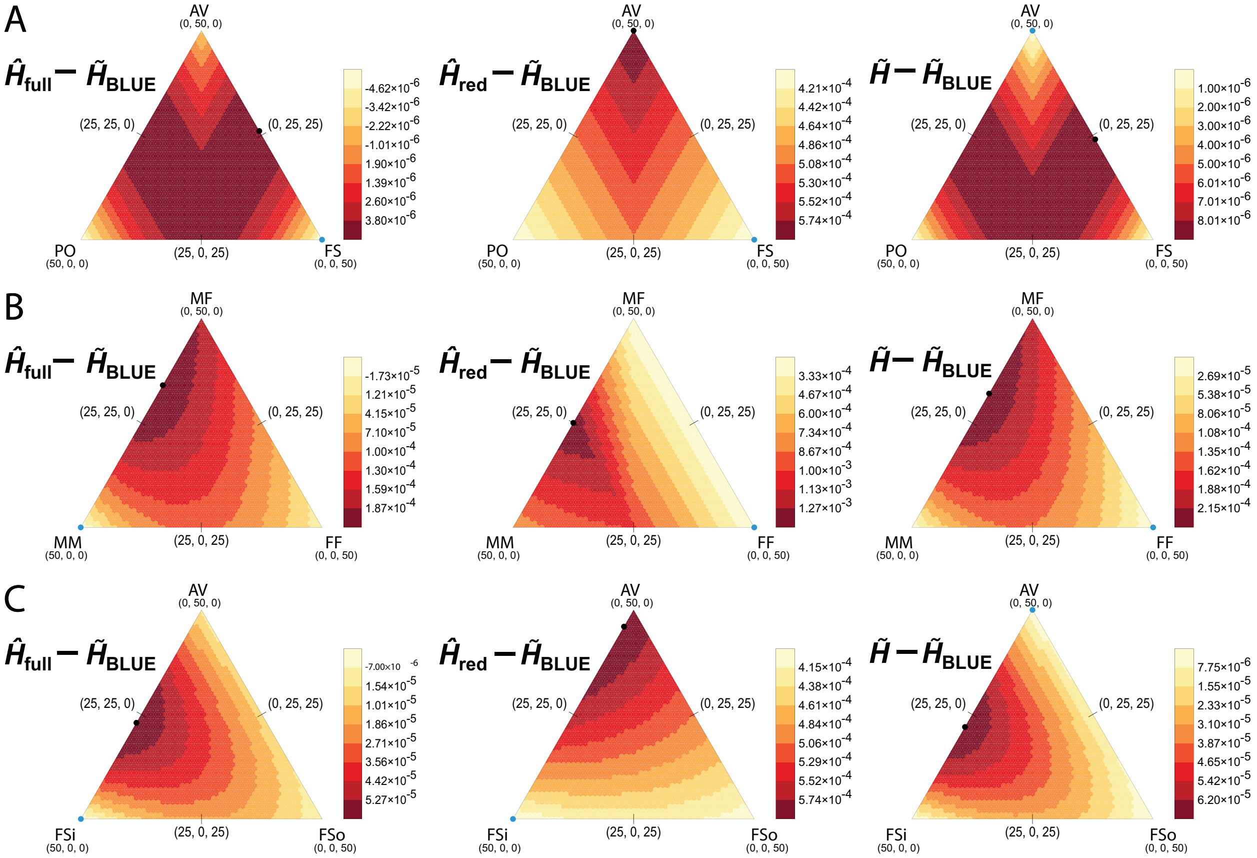

Figure S11: Theoretical difference in mean squared error between $\widehat{H}_{\text{full}}$ (left), $\widehat{H}_{\text{red}}$ (center), or $\widetilde{H}$ (right), and $\widetilde{H}_{\text{BLUE}}$, for samples of 100 (A) outbred diploid individuals, (B) male and female individuals at an X-linked locus, or (C) diploid individuals wherein some full siblings are inbred with brother-sister parents. The samples and MSE values considered for each subtraction were modeled on a biallelic locus with minor allele frequency of 0.05 ($H = 0.095$). Each sample contained 50 relative pairs such that each individual was related to exactly one other. Each sample configuration is a single point in the space of a heat map defined by three coordinates (each representing the count of a relative pair type). For each configuration, the MSE of $\widetilde{H}_{\text{BLUE}}$ is subtracted from that of the other estimators, yielding a value greater than zero. Samples were composed of one to three relative pair types where the vertex of each heat map represents a sample with only a single relative pair type. The relative pair types were (A) parent-offspring (PO), second-degree avuncular (AV), and full-sibling (FS), (B) male-male (MM), male-female (MF), and female-female (FF) full-sibling such that the number of males and females in each sample is not fixed, or (C) inbred full-sibling (FSi), second-degree avuncular (AV), and outbred full-sibling (FSo). Blue and black points indicate the smallest and largest values, respectively, on each map. Threshold values for coloration are indicated in the scales to the right of each heat map, with smaller values colored lighter. Note that the scales are not identical across heat maps.
