# Supplementary material for: An Unbiased Estimator of Gene Diversity with Improved Variance for Samples Containing Related and Inbred Individuals of any Ploidy
Source: G3 (Bethesda). 2016 Dec 30;7(2):671–91. doi: 10.1534/g3.116.037168 (PMC5295611; doi:10.1534/g3.116.037168)
Supplement: Supplementary file 12 [file 671FileS1.docx]

**File S1:** Expected heterozygosity (*H*) values of the 645 human microsatellite loci from the MS5795 dataset, wherein global mean microsatellite allele frequencies are assumed to be the true values, and *H* values of 50 simulated SNP loci, on which analyses were performed. *H* values are arranged in ascending order for each category. (.xlsx, 22 KB)

Available for download as a .xlsx file at:

http://www.g3journal.org/lookup/suppl/doi:10.1534/g3.116.037168/-/DC1/FileS1.xlsx
